# Bayesian Multinomial Logistic Normal Models through Marginally Latent Matrix-T Processes

**Published:** 2022

**Authors:** Justin D. Silverman, Kimberly Roche, Zachary C. Holmes, Lawrence A. David, Sayan Mukherjee

**Affiliations:** College of Information Science and Technology, Department of Statistics, and Institute for Computational and Data Science, Penn State University, University Park, PA, 16802, USA; Program in Computational Biology and Bioinformatics, Duke University, Durham, NC, 27708, USA; Department of Molecular Genetics and Microbiology, Duke University, Durham, NC, 27708, USA; Department of Molecular Genetics and Microbiology and Center for Genomic and Computational Biology, Duke University, Durham, NC, 27708, USA; Departments of Statistical Science, Mathematics, Computer Science, Biostatistics & Bioinformatics, Duke University, Durham, NC, 27708, USA

**Keywords:** Bayesian Statistics, Multivariate Analysis, Count Data, Microbiome, Gene Expression

## Abstract

Bayesian multinomial logistic-normal (MLN) models are popular for the analysis of sequence count data (*e.g*., microbiome or gene expression data) due to their ability to model multivariate count data with complex covariance structure. However, existing implementations of MLN models are limited to small datasets due to the non-conjugacy of the multinomial and logistic-normal distributions. Motivated by the need to develop efficient inference for Bayesian MLN models, we develop two key ideas. First, we develop the class of Marginally Latent Matrix-T Process (Marginally LTP) models. We demonstrate that many popular MLN models, including those with latent linear, non-linear, and dynamic linear structure are special cases of this class. Second, we develop an efficient inference scheme for Marginally LTP models with specific accelerations for the MLN subclass. Through application to MLN models, we demonstrate that our inference scheme are both highly accurate and often 4–5 orders of magnitude faster than MCMC.

## 1. Introduction

Motivated by the growing need for efficient inference for a wide class of multinomial logistic-normal (MLN) models, in this article we develop two key ideas. First, we introduce the class of Marginally Latent Matrix-T Process (Marginally LTP) models. As the name suggests, Marginally LTP models are defined by a shared canonical marginal form which is a multivariate generalization of Student-t processes (Shah et al., 2014) and allow for non-Gaussian likelihoods. We show that this class is extremely flexible, encompassing many useful models including generalized linear models, generalized Gaussian process models, and generalized dynamic linear models. Second, we develop a general inference scheme for Marginally LTP models (which we term the collapse-uncollapse sampler) with specific accelerations (namely a marginal Laplace approximation) for the subclass of MLN models. Through both simulations and analyses of real datasets using MLN models, we show that our inference schemes are both highly accurate and often 4–5 orders of magnitude faster than MCMC.

MLN models are used for the analysis of compositions measured through multivariate counting. In contrast to multinomial Dirichlet models, MLN models permit both positive and negative covariation between multinomial categories (Aitchison and Shen, 1980). While multinomial logistic-normal topic models have been used in natural language processing for some time (Blei and Lafferty, 2006; Glynn et al., 2019), more recently these models have been adopted for regression and time-series modeling of microbiome data (Grantham et al., 2017; Silverman et al., 2018a; Äijö et al., 2017).

Yet, inference in MLN models is challenging due to lack of conjugacy between the multinomial and the logistic normal. Early work with MLN models used Metropolis within Gibbs samplers (Cargnoni et al., 1997; Billheimer et al., 2001) and could scale to just a small number multinomial categories (*i.e*., less than 5). Recently, Pólya–Gamma data augmentation was proposed as a means of inference in MLN regression by augmenting Pólya–Gamma random variables between the multinomial and logistic normal components of a model. Yet for MLN models, the number of Gibbs sampling steps scales linearly with the number of multinomial categories (Polson et al., 2013). Numerous authors have found this approach too computationally intensive to scale to large multinomial models and have instead developed augmentation methods based on a stick-breaking representation of the multinomial (Linderman et al., 2015; Zhang and Zhou, 2017). However, this stick breaking representation does not maintain the logistic-normal form of the model and is sensitive to the labeling of multinomial categories (Linderman et al., 2015). Most recently, several authors (Silverman et al., 2018a; Äijö et al., 2017; Grantham et al., 2017) have shown that Hamiltonian Monte Carlo (HMC) provides for a more efficient and scalable approach to inference in MLN models. In particular, Grantham et al. (2017) used a HMC within a Gibbs sampler whereas both Silverman et al. (2018a), and Äijö et al. (2017) found that the No-U-Turn-Sampling algorithm provided by the Stan Modeling language (Gelman et al., 2015), provided more scalable inference. However, both these approaches are still limited in the number of categories or samples that they can handle. Silverman et al. (2018a) analyzed approximately 800 samples each with only 10 multinomial categories; Äijö et al. (2017) analyzed 36 multinomial categories but had to run their model over the dataset using a sliding window of 60 samples at a time; and Grantham et al. (2017) analyzed 166 samples and 2662 categories but had to impose low rank structure on the logistic normal model for computational tractability. In this work we show that our inference methods scale to hundreds to thousands of categories and samples and permit inference for a wide variety of models including non-linear regression models (as in Äijö et al. (2017)), dynamic linear models (as in Silverman et al. (2018a)), and linear regression models (as in Grantham et al. (2017)).

The layout of this article is as follows. In Section 2 we introduce a common motivation for the use of MLN models. In Section 3 we introduce the class of Marginally LTP models which encompasses many useful MLN models. In Section 4 we develop inference methods for Marginally LTP Models as well as developing specific acceleration for MLN models. In Section 5 we demonstrate our approaches through extensive simulation studies of MLN models. Finally, in Section 6 and 7 we demonstrate the utility of our approaches by developing both linear and non-linear regression models for microbiome sequence count data. Finally, we close with a discussion in Section 8.

## 2. Multinomial Logistic-Normal (MLN) Models

Our primary motivation in this work was to develop efficient inference for a class of models we refer to as multinomial logistic-normal (MLN) models. Consider a dataset Y consisting of N observations of D-dimensional count vectors; where the counting process for each observation is modeled as multinomial. For example, in the analysis of microbiome data we may consider $Y_{\cdot j}$ to be a count vector with a total of $n_{j}=\sum_{i}Y_{ij}$ counts, representing the number of DNA molecules observed for each of D different bacterial taxa in sample j. Yet, in many such datasets, multinomial count variation is just one source important variation. Consider the task of modeling a hypothetical dataset of N political polls each collected in a different year and each counting the number of polled individuals who identify with one of D different political parties. In such a setting we may wish to develop a model of the form:

$$Y_{\cdot t}\sim \text{Multinomial}\left(n_{t},\pi_{\cdot t}\right)$$

π~f(θ)

where f(θ) represents a time-varying stochastic process with parameters θ. Often, a logistic normal model represents an appealing form for f as it, in contrast to Dirichlet models, allows for both positive and negative covariation between the political parties (Aitchison and Shen, 1980). Furthermore, if ϕ represents a log-ratio transform such as the ${\text{ALR}}_{D}$ transform, with inverse given by:

(1) $${\text{ALR}}_{D}^{-1}\left(\eta_{\cdot j}\right)=\left(\frac{e^{\eta_{1j}}}{1+\sum_{i=1}^{D-1}e^{\eta_{ij}}},\cdots ,\frac{e^{\eta_{(d-1)j}}}{1+\sum_{i=1}^{D-1}e^{\eta_{ij}}},\frac{1}{1+\sum_{i=1}^{D-1}e^{\eta_{ij}}}\right),$$

then we can write a multinomial logistic normal (MLN) model as a multinomial transformed-multivariate normal model:

$$Y_{\cdot t}\sim \text{Multinomial}\left(n_{t},\pi_{\cdot t}\right)$$

$$\pi_{\cdot t}=\phi^{-1}\left(\eta_{\cdot t}\right)$$

$$\text{vec}\left(\eta \right)\sim N\left(\mu ,\Sigma \right).$$

This relationship between the logistic-normal and the multivariate normal demonstrates another appealing property of logistic-normal models: They can often be easily formulated as a transformation of existing multivariate normal models.

In what follows, we develop efficient inference methods for a class of models we term Marginally Latent Matrix-T (Marginally LTP) models. We show that the class of Marginally LTP models encompasses many useful MLN models such as linear regression models, non-linear regression models, and time-series models.

## 3. Modeling Overview

In this section we will introduce Marginally Latent Matrix-T Process (Marginally LTP) models as a flexible class of models capable of describing a wide variety of linear regression, non-linear regression, and time-series models.

### 3.1 Matrix-Normal and Matrix-T, Distributions and Processes

To build the class of Marginally LTP models we first review matrix-normal distributions and processes as well as matrix-t distributions and processes, highlighting properties we will make use of in this article.

#### Matrix-Normal Distribution

The matrix-normal distribution is a generalization of the multivariate normal distribution to random matrices. We describe a random m×n matrix X as being distributed matrix-normal Y~N(M,U,V) if vec(Y)~N(vec(M),V⊗U) where ⊗ denotes the Kronecker product, U is a m×m covariance matrix and V is a n×n covariance matrix.

#### Matrix-Normal Process

We define a stochastic process Y as a matrix-normal process on the set $𝒳={𝒳}^{\left(1\right)}\times {𝒳}^{\left(2\right)}$ and denoted Y~GP(M,K,A) if Y evaluated on any two finite subsets $x^{\left(1\right)}=\left(x_{1}^{\left(1\right)},\ldots ,x_{P}^{\left(1\right)}\right)\in {𝒳}^{\left(1\right)}$ and $x^{\left(2\right)}=\left(x_{1}^{\left(2\right)},\ldots ,x_{N}^{\left(2\right)}\right)\in {𝒳}^{\left(2\right)}$ is distributed as Y~N(M,K,A) where $M_{ij}=\text{M}\left(x_{i}^{\left(1\right)},x_{j}^{\left(2\right)}\right),K_{ij}=\text{K}\left(x_{i}^{\left(1\right)},x_{j}^{\left(1\right)}\right),A_{ij}=\text{A}\left(x_{i}^{\left(2\right)},x_{j}^{\left(2\right)}\right)$ for matrix function M and kernel functions K and A. The requirement that K and A be kernel functions implies that the matrices K and A are covariance matrices (*i.e*., they are symmetric positive definite).

#### Matrix-t Distribution

The matrix-t distribution is a generalization of the multivariate-t distribution to random matrices. Like the multivariate-t, the matrix-t can be defined constructively through its relationship to the matrix-normal and inverse Wishart distributions. Let Σ denote a random covariance matrix such that Σ~IW(Ξ,v) where Ξ represents a positive semi-definite scale matrix and v>0. Also suppose that X~N(0,I,V). If $CC^{T}=\Sigma$ then the distribution of Y=CX is denoted as matrix-t such that Y~T(v,0,Ξ,V). For a random matrix η~T(v,B,K,A) the log density of η may be written

(2) $$\text{log}\;T_{P\times N}\left(\eta |v,B,K,A\right)=\text{log}\;\Gamma_{P}\left(\frac{v+N+P-1}{2}\right)-\text{log}\;\Gamma_{P}\left(\frac{v+P-1}{2}\right)-\frac{NP}{2}\text{log}\;\pi -\frac{N}{2}\text{log}|K|-\frac{p}{2}\text{log}|A|-\frac{v+N+P-1}{2}\text{log}\left|I_{p}+K^{-1}[\eta -B]A^{-1}[\eta -B{]}^{T}\right|$$

where $\Gamma_{a}(b)$ refers to the multivariate gamma function. These results follows directly from Gupta and Nagar (2018, p. 134).

#### Matrix-t Process

Through analogy to our definition of matrix normal processes, we define a matrix-t process through its relationship to the matrix-t distribution. We define a stochastic process Y~TP(v,B,K,A) defined on the set $𝒳={𝒳}^{\left(1\right)}\times {𝒳}^{\left(2\right)}$ as a matrix-t process if Y evaluated on any two finite subsets ${\text{x}}^{\left(1\right)}=\left(x_{1}^{\left(1\right)},\ldots ,x_{P}(1)\right)\in {𝒳}^{\left(1\right)}$ and $x^{\left(2\right)}=\left(x_{1}^{\left(2\right)},\ldots ,x_{N}^{\left(2\right)}\right)\in {𝒳}^{\left(2\right)}$ is distributed as Y~T(v,B,K,A) where v is a scalar strictly greater than zero, $B_{ij}=\text{B}\left(x_{i}^{\left(1\right)},x_{j}^{\left(2\right)}\right),K_{ij}=\text{K}\left(x_{i}^{\left(1\right)},x_{j}^{\left(1\right)}\right)$, and $A_{ij}=\text{A}\left(x_{i}^{\left(2\right)},x_{j}^{\left(2\right)}\right)$ for matrix function B, and kernel functions K and A. Matrix-t processes can be alternatively seen as a multivariate generalization of Student-t processes which have found widespread use in statistical analysis (Shah et al., 2014).

### 3.2 Latent Matrix-t Processes (LTPs)

To generalize matrix-t processes to a more flexible set of data types, e.g., count data, we now define LTPs as a generalization of a matrix-t processes. We accomplish this by defining a stochastic process Y as a hierarchical process formed by a process F having parameters that, with appropriate transformation ϕ, follow a matrix-t process. Additionally, we now explicitly denote dependence on model hyper-parameters which we collectively refer to as δ.

**Definition 1 *Latent Matrix*-*t Process*** *We define a stochastic process* Y *as a latent matrix*-*t process* Y~LTP(F,ϕ,v,B,K,A,δ) *on the set* $𝒳={𝒳}^{\left(1\right)}\times {𝒳}^{\left(2\right)}$ *if* Y *evaluated on any* P *dimensional finite subset* $x^{\left(1\right)}\in {𝒳}^{\left(1\right)}$ *and any* N *dimensional finite subset* $x^{\left(2\right)}\in {𝒳}^{\left(2\right)}$ *is distributed*

(3) Y~f(π,δ)

(4) $$\pi =\phi^{-1}(\eta )$$

(5) $$\eta \sim T\left(v,B\left(\delta \right),K\left(\delta \right),A\left(\delta \right)\right).$$

*where* η *denotes a* P×N *real valued matrix,* B(δ) *a* P×N *dimensional real valued matrix function of parameters* δ *defined by* $\left[B(\delta ){]}_{ij}=\text{B}\left(x_{i}^{\left(1\right)},x_{j}^{\left(2\right)},\delta \right),K(\delta \right)$ *is a* P×P *covariance matrix defined as* $\left[K(\delta ){]}_{ij}=\text{K}\left(x_{i}^{\left(1\right)},x_{j}^{\left(1\right)},\delta \right),A(\delta \right)$ *is an* N×N *covariance matrix defined as* $[A(\delta ){]}_{ij}=\text{A}\left(x_{i}^{\left(2\right)},x_{j}^{\left(2\right)},\delta \right),v$ *is a scalar subject to* v>0,π *is an element of a space* Π *defined via the one*-*to*-*one mapping* $\phi^{-1}:{ℛ}^{P\times N}\to \Pi$, *and* f *denotes a probabilistic model for the observed data (a likelihood model), with parameters* π *and* δ, *which is itself an evaluation of the process* F *evaluated on a finite subset of the set Π*.

### 3.3 Marginally LTP Models

To allow us to represent latent processes beyond LTPs, we next introduce a generalization of LTPs to a larger class which we term Marginally LTP models. This definition is straightforward, we define Marginally LTP models as those models which have a marginal that is an LTP.

**Definition 2 *Marginally LTP models*** *If a model described by the joint distribution* p(η,Ψ,Y) *may be written as* p(Ψ∣η,Y)p(η,Y) *where* p(η,Y) *is an LTP, we refer to* p(η,Ψ,Y) *as a Marginally LTP model and* p(η,Y) *as the model’s collapsed representation*.

In the next three subsections we demonstrate that Marginally LTP models provide a rich class of models. We give three examples of Marginally LTP models: (1) a class of multivariate generalized linear models; (2) a flexible class of models for inference in multivariate non-Gaussian time-series; and (3) a flexible class of multivariate generalized non-linear regression models.

#### 3.3.1 Generalized Multivariate Conjugate Linear (GMCL) Models

First we develop generalization of Bayesian multivariate linear regression with conjugate priors which permits non-Gaussian observations (Rossi et al., 2012, p. 32). As in Section 2, let us consider Y to represent N independent D-variate measurements and consider X to represent N sets of Q-dimensional covariates. We define generalized multivariate conjugate linear (GMCL) models as

(6) $$Y_{\cdot j}\sim f\left(\pi_{\cdot j}\right)$$

(7) $$\pi_{\cdot j}=\phi^{-1}\left(\eta_{\cdot j}\right)$$

(8) $$\eta_{\cdot j}\sim N(\Lambda {X.}_{j},\Sigma )$$

(9) Λ~N(Θ,Σ,Γ)

(10) $$\Sigma \sim IW\left(\Xi ,v\right).$$

We may describe the joint density of this model as p(Λ,Σ,η,Y) which can be factored as p(Λ,Σ∣η,Y)p(η,Y). Therefore, to parallel to the definition of Marginally LTP models we may equate Ψ={Λ,Σ}. In Appendix A we prove that p(η,Y) is an LTP with parameters

B=ΘX

K=Ξ

$$A=I_{N}+X^{T}\Gamma X$$

and with {Θ,Γ,Ξ}∈δ. This result demonstrates that all GMCL models are Marginally LTP models. Further, by letting f denote the multinomial distribution and $\phi^{-1}$ denote the inverse ALR transform, we can build multinomial logistic-normal linear models as a special case of GMCL models.

#### 3.3.2 Generalized Multivariate Dynamic Linear Models (GMDLMs)

We develop a flexible class of multivariate time-series models for non-Gaussian observations. We term this class of models generalized multivariate dynamic linear models (GMDLMs). GMDLMs represent an extension of the multivariate dynamic linear models introduced in Quintana and West (1987) and developed further in West and Harrison (1997, Ch. 16) to non-Gaussian observations. Using notation from West and Harrison (1997, Ch. 16), let $\eta_{t}^{T}$ denote a row-vector (*i.e*., the transpose of the t-th column of η). We define the GMDLM as

(11) $$Y_{\cdot j}\sim f\left(\pi_{\cdot j}\right)$$

(12) $$\pi_{\cdot j}=\phi^{-1}\left(\eta_{\cdot j}\right)$$

(13) $$\eta_{t}^{T}=F_{t}^{T}\Theta_{t}+\nu_{t}^{T},\;\nu_{t}\sim N\left(0,\gamma_{t}\Sigma \right)$$

(14) $$\Theta_{t}=G_{t}\Theta_{t-1}+\Omega_{t},\;\Omega_{t}\sim N\left(0,W_{t},\Sigma \right)$$

(15) $$\Theta_{0}\sim N\left(M_{0},C_{0},\Sigma \right)$$

(16) Σ~IW(Ξ,v)

where $\Theta_{t}$ represents a Q×P matrix describing the state of the time-series at time $t,G_{t}$ denotes the Q×Q state transition matrix at time $t,F_{t}$ denotes a Q×1 vector describing a linear model relating the latent space to the parameters $\eta_{t},\Sigma$ is a P×P covariance matrix specifying the covariation between the P dimensions of the time-series, $W_{t}$ is a Q×Q covariance matrix describing the covariation of the perturbations affecting latent states, and $\gamma_{t}$ is a scalar allowing an analyst to weight the importance of select observations ($\gamma_{t}$ is typically equal to 1).

The joint model for the GMDLM can be written p(Θ,Σ,η,Y) which can be factored as p(Θ,Σ∣η,Y)p(η,Y). To parallel the definition of Marginally LTP models, here we have Ψ={Θ,Σ}. In Appendix B we prove that p(η,Y) is an LTP with parameters

$$B=\left[\begin{array}{ccccc} | & \; & | & \; & |\\ \alpha_{1} & \cdots & \alpha_{t} & \cdots & \alpha_{T}\\ | & \; & | & \; & | \end{array}\right]$$

$$\alpha_{t}={\left(F_{t}^{T}{𝒢}_{t:1}M_{0}\right)}^{T}$$

K=Ξ

$$A_{t,t-k}=\{\begin{array}{l} \gamma_{t}+F_{t}^{T}\left[W_{t}+\overset{2}{\underset{\ell =t}{\sum }}{𝒢}_{t:\ell }W_{\ell -1}{𝒢}_{\ell :t}^{T}+{𝒢}_{t:1}C_{0}{𝒢}_{1:t}^{T}\right]F_{t}\;\text{if}\;k=0\\ F_{t}^{T}\left[{𝒢}_{t:t-k+1}W_{t-k}+\overset{2}{\underset{\ell =t-k}{\sum }}{𝒢}_{t:\ell }W_{\ell -1}G_{\ell :t-k}^{T}+{𝒢}_{t:1}C_{0}G_{1:t-k}^{T}\right]F_{t-k}\;\text{if}\;k>0 \end{array}$$

where we have introduced ${𝒢}_{t:\ell }$ as a short hand notation for the product $G_{t}\cdots G_{\ell }$ and where we have hyper-parameters $\left\{\Xi ,M_{0},C_{0},W_{1},\ldots ,W_{T},\gamma_{1},\ldots ,\gamma_{T},G_{1},\ldots ,G_{T},F_{1},\ldots ,F_{T}\right\}\in \delta$. This result demonstrates that GMDLMs are a special case of Marginally LTP models.

### 3.4 Generalized Multivariate Gaussian Process (GMGP) Models

Finally, we develop a flexible class of generalized multivariate non-linear models based on the matrix normal processes discussed in Section 3.1. These models utilize a separable kernel structure to allow modeling of vector valued data as seen, for example, in coregionalization models Álvarez et al. (2012). As a motivating example, suppose that we wish to model a microbiome time-series. In particular, suppose we wish to predict the relative abundance of an unobserved taxa at an unobserved time-point. Let us consider X to encompass available temporal metadata for observed samples, e.g., time-indices as well as other relevant covariates influencing composition at each observed time-point. Further, let us consider Z to encompass available metadata regarding each observed bacterial taxa, e.g., 16S sequence as well as whether the bacteria is aerobic or anaerobic. In this section we describe a flexible class of models which we term Generalized Multivariate Gaussian Process (GMGP) models which are capable of performing this, as well as many other, analysis tasks.

To enable GMGP models to make predictions regarding unobserved multinomial categories (e.g., unobserved taxa) we must first define Inverse Wishart Processes. These processes can be defined constructively in a similar manner to the matrix normal and matrix-t processes we defined in Section 3.1. Given a set Z with P-dimentional finite subset $Z=\left[Z_{\cdot 1},\ldots ,Z_{\cdot P}\right]$, a scalar ν>0, and a kernel function Ξ such that $\Xi_{ij}=\Xi \left(Z_{\cdot i},Z_{\cdot j}\right)$, we define a stochastic process Σ~IWP(Ξ,ν) as an Inverse Wishart Process on the set Z if Σ evaluated on any subset Z is distributed as Σ~IW(Ξ,ν+p). In words, an Inverse Wishart Process is a probability distribution over kernel functions.

Using the above construction of Inverse Wishart Processes, we can now define the GMGP model form:

(17) $$Y_{\cdot j}\sim f\left(\pi_{\cdot j}\right)$$

(18) $$\pi_{\cdot j}=\phi^{-1}\left(\eta_{\cdot j}\right)$$

(19) $$\eta_{\cdot j}\sim N\left(\Lambda \left(X_{\cdot j}\right),\Sigma \left(Z\right)\right)$$

(20) Λ~GP(Θ,Σ,Γ)

(21) Σ~IWP(Ξ,ν)

where Θ is a mean function and Γ as well as Ξ are kernel functions.

We may describe the joint density of the above model as p(Λ,Σ,η,Y,X) which can be factored as p(Λ,Σ∣η,Y,X)p(η∣Y,X). In Appendix C, we prove that p(η∣Y,X) is an LTP with parameters B=Θ,K=Ξ, and A=I+Γ where I represents the identity kernel defined by:

$$\text{I}\left(x_{i},x_{j}\right)\{\begin{array}{ll} 1 & \text{if}\;x_{i}=x_{j}\\ 0 & \text{otherwise} \end{array}.$$

It should be noted that the LTP form of GMGP models is very similar to that of GMCL models; the major difference between GMGP and GMCL models being the use of mean and kernel functions in place of mean and covariance matrices. Still, we discuss these models separately as they will often be used in very different ways – GMCL models for inferring linear effects of covariates, GMGP models for non-linear smoothing and prediction. We demonstrate examples of both of these models using real data in Sections 6 and 7.

## 4. Inference in Marginally LTP Models

Our overarching goal was to develop efficient and accurate posterior inference for MLN models, many of which are a special case of Marginally LTP models. In this section, we demonstrate how the canonical LTP form of Marginally LTP Models can be exploited for efficient inference of this larger model class. types of parameters, η which are distributed matrix-t and of a model to produce a LTP form. The sampling η on η and observed data (p(Ψ∣η,Y)). In Section 4.1 we introduce a sampling scheme for Marginally LTP models which we refer to as the collapse-uncollapse (CU) sampler which exploits the hierarchical structure of Marginally LTP models to improve computational efficiency for various types of inference. In Section 4.2 we further build on the CU sampler by introducing a Laplace approximation as a means of accelerating a bottleneck step in the CU sampler. In Sections 4.3 we discuss the CU sampler in the context of the GMCL, GMDLM and GMGP models introduced in the last section. In Section 4.4, we discuss error bounds for the Laplace approximation. In Section 4.5, we discuss inference of hyperparameters. Finally, in Section 4.6 we discuss the *fido* software package which implements a number of MLN models using the CU sampler with Laplace approximation based on these models Marginally LTP form.

### 4.1 The Collapse-Uncollapse (CU) Sampler

Consider the task of sampling from the posterior distribution of a Marginally LTP model with joint density p(Ψ,η,Y). The corresponding posterior density can be decomposed as

$$p(\eta ,\Psi |Y)=p(\Psi |\eta ,Y)\frac{p(\eta ,Y)}{p(Y)}.$$

This decomposition implies that, given a Marginally LTP model with joint probability p(η,Ψ,Y), we may sample from the posterior by first sampling from the posterior of the collapsed (LTP) model p(η,Y) and then given that sample of η and the observed Y we may then sample Ψ from the conditional p(Ψ∣η,Y). Together the sample of η and Ψ then represents a single sample from the posterior of the Marginally LTP model, p(Ψ,η∣Y) (Algorithm 1).


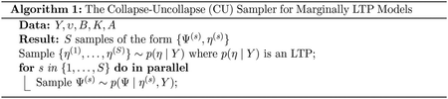


Our rationale for focusing on the CU sampler for inference in Marginally LTP models is as follows. We expect that many Marginally LTP models (such as those introduced in Section 3) have partial conjugacy. Exploiting this partial conjugacy is central to many popular methods such as Metropolis-within-Gibbs (Cargnoni et al., 1997). Yet, by embedding MCMC steps within a Gibbs sampler techniques such as adaptation (Gelman et al., 2015) or approximate methods such as Laplace approximations may not make sense as they would have to be recomputed at each step. In contrast, the CU sampler allows the non-conjugate sampling to occur up front (in the sampling of p(η∣Y)) so that such techniques can be used. Moreover, after multiple samples of η have been produced, uncollapsing the model can be done in parallel for each sample of η. Therefore, the CU sampler may be advantageous as it permits the use of adaptive or approximate methods for sampling the non-conjugate model components and permits a degree of parallelism not allowed by Metropolis-within-Gibbs.

The CU Sampler for Marginally LTP Models therefore requires two features for efficient inference. First, we require an efficient means of producing samples from the collapsed (LTP) form p(η∣Y). As we will show in Section 5, sampling p(η∣Y) can be more efficient than sampling p(Ψ,η∣Y) just by virtue of the fact that the former has fewer dimensions. Therefore the CU sampler alone can be more efficient than sampling the full (uncollapsed) model. Still, in Section 4.2 we develop a Laplace approximation for p(η∣Y) which can further improve efficiency. Second, we require an efficient means of sampling from the posterior conditional p(Ψ∣η,Y). In Section 4.3 we discuss efficient means of sampling p(Ψ∣η,Y) for the GMCL, GMDLM, and GMGP model classes.

### 4.2 Laplace Approximation for the Collapsed Form

Sampling p(η∣Y) is often the major computational bottleneck when inferring Marginally LTP models via the CU sampler. To accelerate this step, we developed a Laplace approximation for the density p(η∣Y). This approximation is defined as $q(\eta |Y)=N\left(\text{vec}\;\overset{ˆ}{\eta },H^{-1}(\text{vec}\;\overset{ˆ}{\eta })\right)$ where $\overset{ˆ}{\eta }$ denotes the *maximum a posteriori* (MAP) estimate of p(η∣Y) and $H^{-1}(\text{vec}\;\overset{ˆ}{\eta })$ denotes the inverse Hessian matrix of logp(η∣Y) evaluated at the point vec $\overset{ˆ}{\eta }$. That is, $\overset{ˆ}{\eta }$ is defined as the solution to the following optimization problem

(22) $$\overset{ˆ}{\eta }=\underset{\eta \in {ℛ}^{P\times N}}{\text{argmin}}\left[-\text{log}\;p\left(\eta |Y\right)\right].$$

The solution to this optimization problem is discussed in Appendix F.

While the accuracy of our Laplace approximation will depend on a number of factors including the choice of likelihood, prior, and link function, we hypothesized that such a Laplace approximation would provide an accurate approximation to an LTP posterior in a number of common settings. First, all exponential family likelihoods are log-convex with respect to their natural parameters (Jordan, 2010). Therefore, we expect the Laplace approximation to be particularly useful with any choice of likelihood f from the exponential family (e.g., the multinomial distribution) and with a corresponding choice of ϕ such that η represents the natural parameters of f (e.g., the ALR transform). Second, with regards to the matrix-t prior, the matrix-normal can provide a good approximation for the matrix-t for suitably large v (Gupta and Nagar, 2018, p. 137) as it is both globally symmetric and log-convex about the MAP estimate. We hypothesized that even though the matrix-t is not globally log-convex except as v→∞, in practice the log-convexity about the MAP estimate coupled with its global symmetry would be enough to provide a useful approximation even for small values of v. We note that both our simulation studies in Section 5 and analyses of real data in Section 6 suggest this hypothesis is reasonable. Finally, specifically for models parameterized by probabilities (such as the Multinomial logistic-normal), MacKay (1998) showed that the softmax parameterization can produce more accurate Laplace approximations than the more traditional simplex basis. Notably, the inverse ALR parameterization we choose is a linear transformation of the softmax transform (Pawlowsky-Glahn et al., 2015) and therefore has identical accuracy to a Laplace approximation using the softmax parameterization (MacKay, 1998). Together, these features led us to hypothesize that a Laplace approximation could provide a useful and accurate approximation for the the posterior of an LTP.

Developing an efficient Laplace approximation for LTP models required closed form solutions for the gradient and Hessian of LTPs. To develop these tools note that, by Bayes rule, we may write

(23) $$-\text{log}\;p(\eta |Y)\propto -\text{log}\;f\left(Y|\phi^{-1}(\eta )\right)-p(\eta ).$$

By linearity of the derivative operator we may write the gradient and Hessian of -logp(η∣Y) as

(24) $$-\frac{d\;\text{log}\;p(\eta |Y)}{d\text{vec}(\eta )}=-\frac{d\;\text{log}\;f\left(Y|\phi^{-1}(\eta )\right)}{d\text{vec}(\eta )}-\frac{d\;\text{log}\;p(\eta )}{d\text{vec}(\eta )}$$

(25) $$-\frac{d^{2}\;\text{log}\;p\left(\eta |Y\right)}{d\text{vec}\left(\eta \right)d\text{vec}{\left(\eta \right)}^{T}}=-\frac{d^{2}\;\text{log}\;f\left(Y|\phi^{-1}\left(\eta \right)\right)}{d\text{vec}\left(\eta \right)d\text{vec}{\left(\eta \right)}^{T}}-\frac{d^{2}\;\text{log}\;p\left(\eta \right)}{d\text{vec}\left(\eta \right)d\text{vec}{\left(\eta \right)}^{T}}.$$

Thus we find that calculating the gradient and Hessian of LTPs reduces to calculating the gradient and hessian of $\text{log}\;f\left(Y|\phi^{-1}(\eta )\right)$ and the matrix-t density logp(η∣X) separately. The additive structure of the gradient and Hessian are central to generalizing this approach to a variety of different observation distributions f and transformations $\phi^{-1}$. In Appendix D we provide the gradient and Hessian for the matrix-t density. With these results, to derive a flexible class of multinomial logistic-normal models, we only need to provide the gradient and Hessian for the logit-parameterized multinomial which we give in Appendix E. We describe the implementation of the Laplace Approximation for an LTP in Appendix F.

### 4.3 Efficient Sampling from Posterior Conditionals

The second step of the CU sampler involves sampling from the density p(Ψ∣η,Y). While the density of p(Ψ∣η,Y) is specific to the particular Marginally LTP model, we develop efficient means of sampling from this density for the GMCL, GMDLM, and GMGP models in Appendices A, B, and C respectively. In particular, for all three of these model classes we make use of the fact that Ψ is conditionally independent of Y given η, that is p(Ψ∣η,Y)=p(Ψ∣η). This conditional independence also reduces sampling from the conditionals to computing the posterior distribution of standard Bayesian multivariate linear regression for GMCL and GMGP model and conjugate multivariate dynamic linear models for the GMDLM model. That is, for all three of these model classes, sampling the conditionals reduces to posterior inference for equivalent Bayesian Gaussian models that have been well described previously and have efficient closed form solutions.

### 4.4 Error Rate for a Laplace Approximation to the Collapsed Form

The inference scheme we propose above for Marginally LTP models has two parts: First, sample from the collapsed LTP representation of the model; Second, uncollapse those samples to produce samples from the full Marginally LTP model. If, as we discuss above, we use a Laplace approximation to sample from the collapsed LTP representation, then the only error induced by this inference scheme is due to the Laplace approximation. We wanted to develop intuition regarding the error rate of this approximation when the observation distribution is a logit-parameterized multinomial. In Appendix K, we prove that for large v this error rate is $O_{p}\left((D-1)\sum_{j=1}^{N}n_{j}^{-1}\right)$. That is, the error is stochastically bounded by the sum of the inverse of the average number of counts in each sample. This result follows from theory recently proposed by Ogden (2018) and provides a more general error bound than those used by Kass and Steffey (1989) or Rue et al. (2009). In particular, this bound accounts for not only the number of observed multinomial samples (N) but the number of counts in each multinomial sample $\left(n_{j}\right)$ and the dimension over which those counts are spread (D).

This error bound is intuitive in a number of ways. First, a multinomial sample j with $n_{j}$ counts can be thought of as $n_{j}$ independent observations; it is therefore intuitive that our error bound is proportional to $n_{j}^{-1}$. Second, the number of dimensions in the Laplace approximation to a multinomial sample grows linearly with one minus the number of multinomial categories; intuitively, our error bound is proportional to D-1. Third, the number of dimensions in the Laplace approximation to the collapsed LTP form grows linearly with the number of observed samples; intuitively, our error bound grows (approximately) linearly with the number of observed samples. Finally, based on the observation that the multinomial parameterized by log-ratio coordinates is globally log-convex (Jordan, 2010) whereas the matrix-t distribution is only log-convex near the mean; it makes intuitive sense that a stronger likelihood (implied by larger values of $n_{j}$) would decrease the error of the Laplace Approximation.

This error bound also sheds light on when this Laplace approximation in the CU sampler will provide a useful, accurate inference method for MLN models. For example, this error bound suggests that an ideal dataset for this Laplace approximation is one that has many non-zero counts and lower data-sparsity. In contrast, it suggests that the Laplace approximation should not be used for high-dimensional classification problems, where there are many multinomial categories but only a single non-zero entry per sample. Still, as we demonstrate in the next section, the Laplace approximation can handle substantial data sparsity and many small counts with only minimal error.

### 4.5 Hyperparameter Inference

Until this point we have not considered the presence of unknown hyperparameters in the LTP form (i.e. we have considered δ or ν as given). Yet, for a number of Marginally LTP models, we expect estimation of such hyperparameters will be of interest. For example, within the GMDLM model we anticipate researchers may want to allow the terms $W_{t}$ to be subject to their own stochastic model. This would in turn require that some portion of δ is unknown. Alternatively, for GMCL models, analysts may want to infer the degree-of-freedom parameter ν empirically rather than setting it based on subjective prior information. Overall, we leave inference of ν and δ as future work but note a few potential avenues for practitioners looking to infer these parameters. When the hyper-parameter set {ν,δ} is small, these parameters may be efficiently selected by cross-validation (Rasmussen, 2003). In contrast, when the set is large (i.e., when δ is high-dimensional), alternative approaches are likely needed. In particular, we note that Type-II MAP estimation can provide an efficient means of empirically setting hyper-parameters in a variety of hierarchical Bayesian models (Riihimäki et al., 2014).

### 4.6 Software for Marginally LTP models with Multinomial Observations

For inference of Marginally LTP models with multinomial observations and log-ratio link functions, we developed the R package *fido* (Silverman, 2019). *Fido* implements the CU sampler with Laplace approximation described above using optimized C++ code. Estimation of $\overset{ˆ}{\eta }$ is performed using the L-BFGS optimizer which we have found provides efficient and stable numerical results. Additionally all code required to reproduce the results of the next two sections, including the alternative implementations of multinomial logistic-normal linear models discussed in Section 5 is available as a GitHub repository at github.com/jsilve24/fido_paper_code.

## 5. Simulations

We performed a series of simulation studies to evaluate the CU sampler both with and without the Laplace Approximation in terms of accuracy and efficiency of posterior inference of multinomial logistic-normal models. The only portion of our inference algorithm that is approximate is the Laplace approximation to the LTP form. As this form is shared by all marginally LTP models, we focus our simulations only on multinomial logistic-normal linear models for simplicity (*e.g*., Equations (6)–(10) where f is the multinomial distribution and ϕ is the ${\text{ALR}}_{D}$ transform). To evaluate the utility of the CU sampler we compared Hamiltonian Monte Carlo (HMC) of the full model (HMC Uncollapsed) to the CU sampler where sampling of the collapsed (LTP) form was performed using HMC (HMC Collapsed). Both HMC implementations were inferred using the highly optimized No-U-Turn-Sampler provided in the Stan modeling language (Gelman et al., 2015) which has been frequently used for the analysis of MLN models (Äijö et al., 2017; Silverman et al., 2018a). To further evaluate the utility of the Laplace approximation to the collapsed form in the CU sampler (LA Collapsed), we used the function *pibble* from the *fido* software package described in Section 4.6. Finally, to compare LA Collapsed to an alternative scheme for approximate inference, we included two mean-field automatic-differentiation Variational Bayes (VB) implementations (Kucukelbir et al., 2015). The first was a VB approximation to the full form (VB Uncollapsed), the second was a VB approximation to just the collapsed form of the CU sampler (VB Collapsed). VB Uncollapsed was unstable in practice and often resulted in error during optimization (likely due to the increased number of parameters in the uncollapsed model). As a result, only the results form VB Collapsed could be shown below.

**Figure 1:**
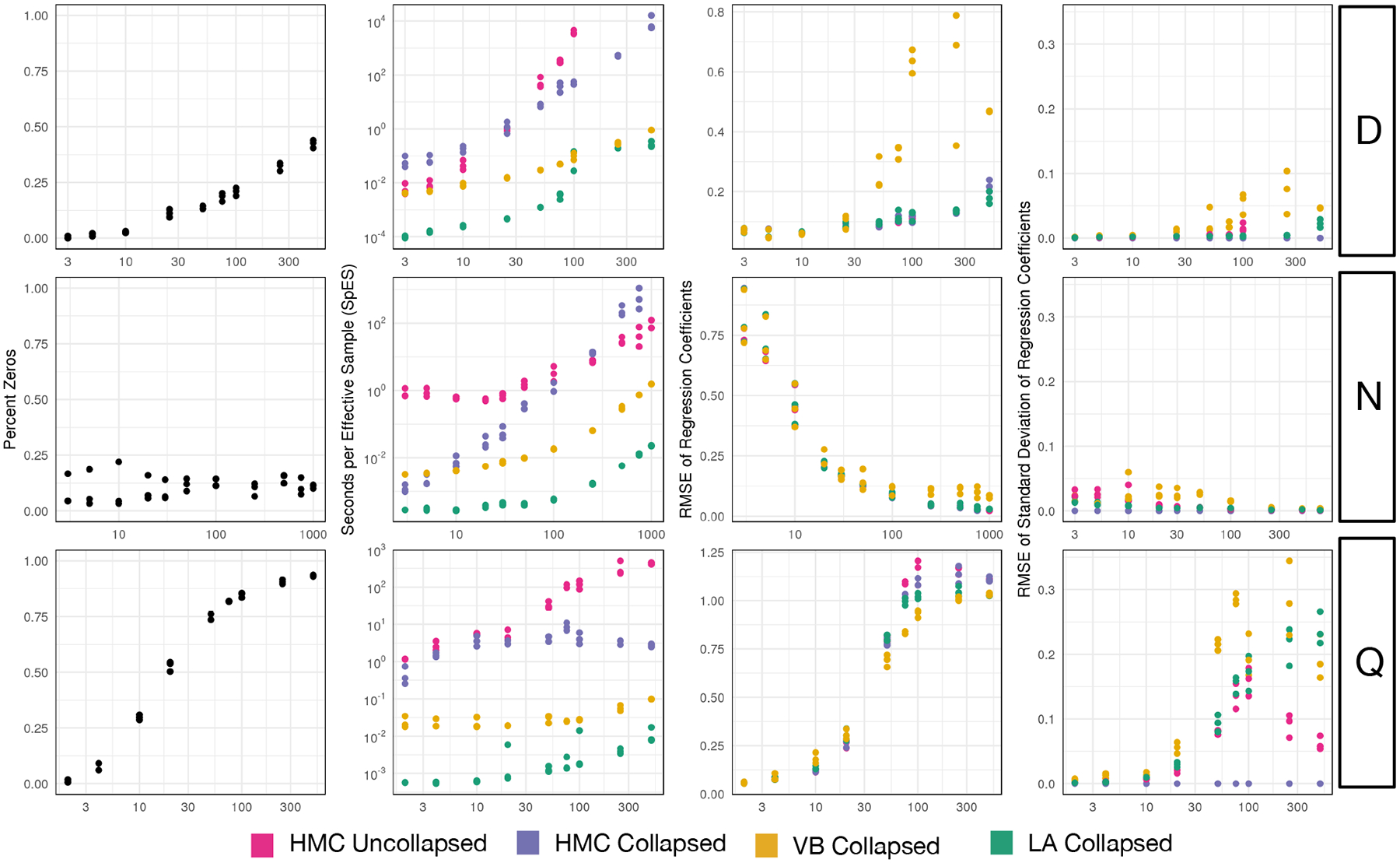
Simulation study comparing multinomial logistic normal linear model implementations. Each row of plots depicts simulation results for varying a different simulation parameter (D, the number of multinomial categories; N, the number of samples; and Q, the number of covariates). The percent of counts that were zero in each simulation is given in the first column. The error bound of the Laplace approximation, which was developed in Section 4.4, is shown in Figure S1. Implementations were compared in terms of efficiency (measured SpES), accuracy of point estimation (measured by RMSE of Regression Coefficients), and accuracy of uncertainty quantification (measured by RMSE of Standard Deviation of Regression Coefficients). For VB Collapsed and LA Collapsed, the number of effective samples is taken to be equal to the total number of samples as both methods produce independent samples from an approximation to the posterior.

In order to compare these implementations, we created a series of simulations based on the corresponding likelihood model (Appendix H). We identified three key parameters, the sample size (N), the observation dimension (D), and the number of model covariates (Q) which we varied in order to test each implementation over a wide range of conditions. By varying these parameters in different simulations we were able to vary the error bound for the Laplace approximation introduced in Section 4.4 (Figure S1). We designed these simulations to span a wide range of sparsity (Figure 1, Column 1). We choose the tuple {N=100,D=30,Q=5} as our base condition and independently varied each simulation parameter from that base condition (N from 10 to 1000, D from 3 to 500, and Q from 2 to 500). Each panel in Figure 1 shows a different simulation metric (*e.g*., percent of data matrix Y that were zero counts or the performance of a given inference method on each simulation) for a given tuple when a particular element of the tuple (N,D,Q) is varied from the base condition. For example, the top left panel shows the sparsity of each dataset for N=100,Q=5, and where D is varied (x-axis). Additionally, to account for the stochastic nature of the simulations, three simulations were performed for each tuple {N,D,Q}. For each simulation, each of the five implementations were fit and allowed a maximum of 48 hours to produce 2000 samples. Prior hyper-parameters were chosen to reflect common default choices, *e.g*., mean parameters set to zero, and covariance parameters set to the identity matrix. The prior degrees-of-freedom parameter ν is defined on the range ν>D, this parameter was chosen as ν=D+10. Further details of the simulation and model fitting procedure can be found in Appendix H.

To quantify the accuracy and efficiency of each implementation we defined the following performance metrics. As a measure of efficiency, we calculated the average number of seconds needed for the implementation to produce one independent sample from the target posterior (*i.e*., Seconds per Effective Sample - SpES). Specifying independent samples is important as HMC samplers produce autocorrelated samples. In contrast, both LA Collapsed and VB Collapsed produce independent samples from the approximate posterior, as a result, for these two methods, SpES equals the number of samples per second. To quantify the accuracy of point estimates from each implementation (*i.e*., either the posterior mean or MAP estimates) we used the root mean squared error of the point estimate for Λ from its true simulated value. Notably, given finite N we do not expect that any implementation will be able to perfectly reconstruct the true simulated value for Λ; rather, this metric provides a means of comparing the relative performance of each implementation. Finally, to quantify the accuracy of uncertainty quantification from each implementation we compared posterior intervals against those of the HMC Collapsed model which was taken as a gold standard. In particular, we define the root mean squared error of standard deviations as the average difference between the estimated posterior standard deviations, $\text{sd}\left(\Lambda_{ij}\right)$, compared to the estimates produced by HMC Collapsed.

Beyond our error bound for the Laplace approximation, we hypothesized that the proportion of zero values in the dataset would impact the accuracy of both the Laplace and variational approximations. In particular, we hypothesized that datasets with higher than 30% zero values would see a substantial degradation in approximation accuracy. As hypothesized we found that the proportion of zero values (the sparsity) of the dataset closely resembled approximation accuracy (Figure 1). Yet, we found that in practice, LA collapsed performed far better than expected: LA Collapsed provided nearly identical estimates of posterior uncertainty to both HMC implementations up to over 90% data sparsity. Additionally, LA Collapsed provided nearly identical point estimates to both HMC implementation over the full spectrum of simulations. Finally, LA Collapsed was often up to 5 orders of magnitude faster than HMC and often 1–2 orders of magnitude faster than VB.

### 5.1 Computational Efficiency

Overall, the CU sampler with a Laplace approximation (LA Collapsed) provided the most efficient inference across all tested conditions. More specifically LA Collapsed displayed speed-ups of between 1 to 5 orders of magnitude in comparison to HMC Collapsed and HMC Uncollapsed and often between 1–2 order of magnitude compared to VB Collapsed. Notably, HMC Uncollapsed failed to complete sampling within 48 hours for D>100.

Beyond the high efficiency of LA Collapsed, our results also demonstrate that the CU sampler can improve inference in HMC without the use of approximate inference methods. These results likely stem from the smaller number of dimensions in HMC for the collapsed versus uncollapsed implementations. Most noticeably, the collapsed representation completely removes dependency on Q from HMC run-times as Λ is marginalized out of the collapsed representation. However, for large N the HMC Uncollapsed is more efficient than HMC Collapsed. This later result may reflect that the heavy tails of the matrix-t distribution produce a more challenging geometry for HMC than the expanded matrix normal and inverse Wishart forms. Such a finding has been well described previously for both univariate and multivariate-t distributions (Stan Development Team, 2018, Section 20).

### 5.2 Point Estimation

Overall point estimation using LA Collapsed (*i.e*., MAP estimates) was nearly identical to point estimation using either HMC Collapsed or HMC Uncollapsed (*i.e*., mean estimates). In contrast, point estimation using VB Collapsed produced substantially larger errors, especially for large values of D. Overall these results demonstrate that the CU sampler maintains accuracy in point estimation and that MAP estimation provides an excellent approximation to the mean in multinomial logistic normal models.

### 5.3 Uncertainty Quantification

Beyond accuracy of point estimates, we also wanted to study the accuracy of estimates of uncertainty from each implementation. We consider the HMC Collapsed implementation to be the gold standard on which we based our performance metric (*RMSE of standard deviations*). Except for values of Q greater or equal to 250 (where the proportion of zero values is >90%), the uncertainty estimates of LA Collapsed were nearly identical to those of both HMC implementations. Yet, at larger values of Q, when sparsity is >90%, we observed differences not only between LA Collapsed and HMC but between the two HMC implementations themselves. There are two possible explanations for this. First, that LA Collapsed had a slightly better point estimation accuracy in these same large Q simulations could point to the fact that LA Collapsed is correct and instead HMC estimates of uncertainty were incorrect due to the often small effective sample size for large Q. Alternatively, this could support our previous hypothesis that the Laplace approximation had higher error in uncertainty quantification with higher data sparsity. Given the ergodicity of HMC it seems more likely that the Laplace approximation is in error in these regions of high sparsity. Yet, that the approximation only began to show substantial error when sparsity is >90% is notable. Beyond LA Collapsed and the HMC implementations, VB Collapsed consistently demonstrated higher error in uncertainty quantification as compared to the other implementations.

Finally, to provide context regarding the size of the differences in uncertainty quantification, we provide direct visualizations of posterior intervals for all four implementations in Figure S3 and S4. These two simulations were chosen to highlight a case in which LA Collapsed was highly accurate (Figure S3) in terms of uncertainty quantification and a case in which it differed from HMC estimates (Figure S4). Notably, visualization of posterior intervals consistently demonstrated that the posterior mean was centered symmetrically in the 95% credible regions. This symmetry suggested that our metric *RMSE of standard deviations* captures much of the discrepancies in uncertainty quantification without higher order moments. Additionally, for context, we include a fifth implementation, PCLM (pseudo-count augmented linear model). The PCLM uses a pseudo-count based estimate of η which ignores the multinomial count variation. Such approximations are common in the analysis of microbiome sequence count data (Silverman et al., 2017; Gloor et al., 2016). Unsurprisingly, this PCLM implementation demonstrated substantially higher error rates than any of the other implementations (Figure S5).

## 6. Identifying Biomarkers of Crohn’s Disease Using Microbiome Data

Crohn’s Disease (CD) is a type of inflammatory bowel disease that has been linked to aberrant immune response to intestinal microbiota (Jostins et al., 2012; Khor and Hibberd, 2012; Gevers et al., 2014). To demonstrate that LA Collapsed (from the R package *fido*) provides an accurate and efficient means of modeling real microbiome data, we reanalyzed a previously published study comparing microbial composition in the terminal ileum of subjects with CD to healthy controls (Gevers et al., 2014). Only LA Collapsed could efficiently scale to this data size (49 taxa, 250 samples, 4 covariates). To allow us to compare to alternative implementations we randomly subset the data to contain 83 samples. On this subset HMC Uncollapsed and VB Collapsed repeatedly failed to run due to numerical instability. In addition, LA Collapsed produced posterior estimates nearly identical to HMC Collapsed but more than 1000 times faster.

Using the four model implementations introduced in Section 5, a Bayesian multinomial logistic normal linear model was fit to investigate the relationship between bacterial composition and CD. For both the full data-set and the subset, our regression model was defined for the j-th sample by the covariate vector

$$x_{j}={\left[1,x_{j(\text{CD})},x_{j(\text{Inflamed)}},x_{j(\text{Age)}}\right]}^{T}$$

where $x_{j(\text{CD})}$ is a binary variable denoting whether the j-th sample was from a patient with CD or a healthy control, $x_{j\text{(Inflamed)}}$ a binary variable denoting inflammation at time of sample collection, $x_{j\text{(Age)}}$ denoting age of the subject, and the preceding 1 represents a constant intercept. To evaluate the impact of using small values for the degree-of-freedom parameter ν in model priors, we set ν=D+3. A full description of our prior assumptions is given in Appendix I and results of posterior predictive checks are shown in Figure S6.

Even though all four implementations were initialized identically, both the HMC Uncollapsed and VB Collapsed implementations repeatedly resulted in errors due to numerical instability. Thus only LA Collapsed and the HMC Collapsed implementations could fit this model for even the subset dataset. Whereas the HMC Collapsed model took approximately 30 minutes, LA Collapsed took only 3 seconds. Thus LA Collapsed is over 1000 times faster than HMC Collapsed on real data. Additionally, posterior estimates of Λ produced by both the HMC Collapsed and LA Collapsed implementations are nearly identical (Figure S7). These results demonstrate that in real data scenarios LA Collapsed can provide efficient and accurate posterior inference.

**Figure 2:**
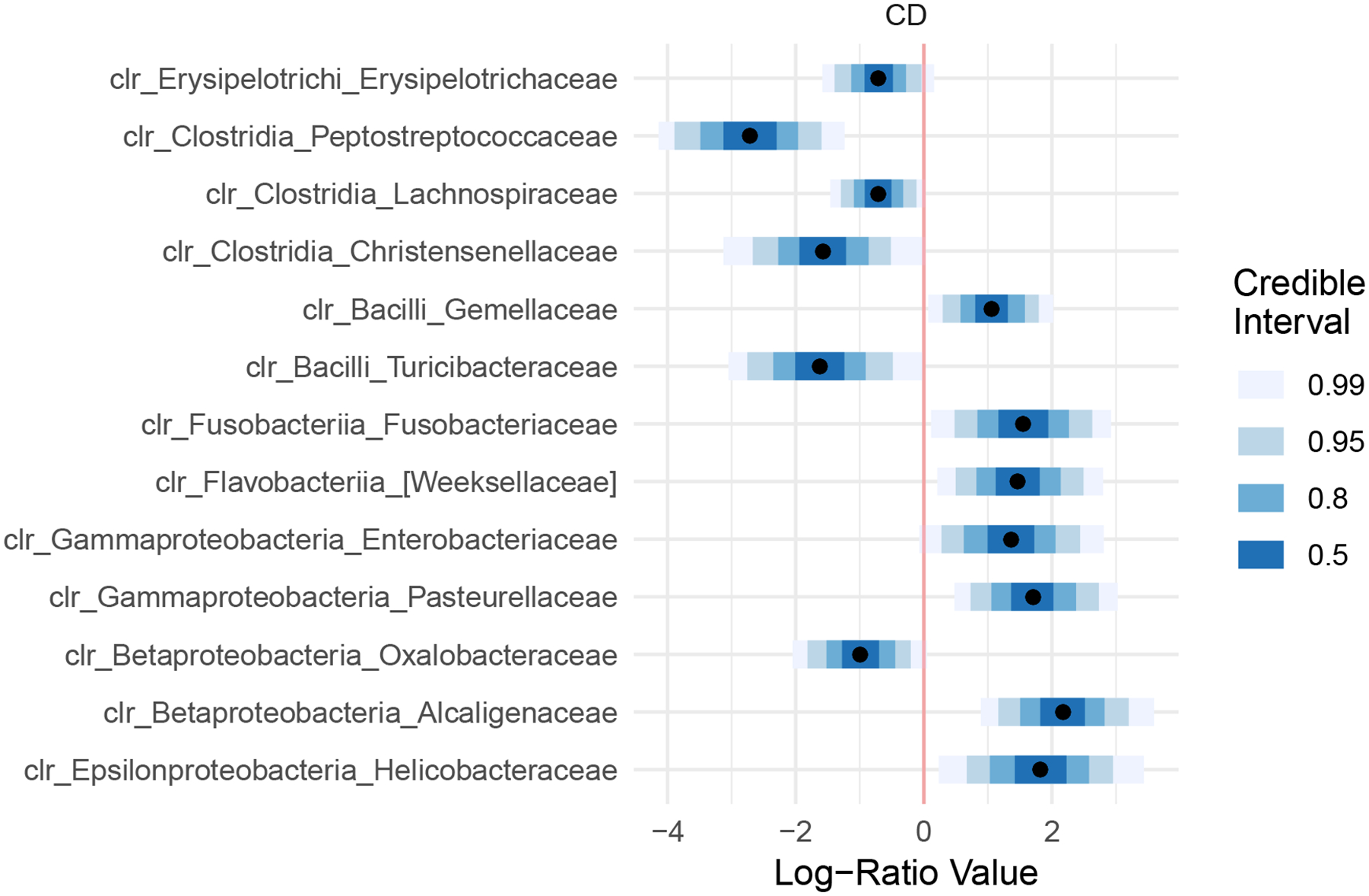
Posterior mean and credible intervals for Λ *of fido::pibble* (LA Collapsed) applied to Crohn’s disease data. Only the 12 families found to be associated with Crohn’s Disease (CD) (*i.e*., Posterior 95% credible region not covering zero) are shown. Taxa are denoted as clr_[class]_[family]. Λ is represented in centered log-ratio (CLR) coordinates rather than additive log-ratio (ALR) so that each coordinate could be identified with a different bacterial taxa.

By modeling the full dataset we found the centered log-ratio (CLR) coordinates corresponding to 12 genera to be associated with CD status (95% credible interval not covering 0; Figure 2). These results are in general agreement with prior analyses (Gevers et al., 2014). As in prior analyses, we find a substantial increase in the abundance of proteobacteria in CD versus healthy controls. Similarly, we find that the families Pasteurellaceae and Enterobacteriacaeae, Gemellaceae, and Fusobacteriaceae are highly enriched and that the class Clostridia are depleted in CD. Notably, Fusobacteria has been independently suggested as a marker of IBD (Strauss et al., 2011; Kostic et al., 2012). These findings serve to validate our results and build confidence in our methods.

In contrast, our results differ from prior analyses of this data in certain respects. We find that the family Peptostreptococcaceae is likely decreased in CD versus healthy controls and we find no association for Veillonellaceae. Three factors support our results. First, our analysis accounts for count variation and compositional constraints whereas prior analyses have not. It is well known that the handing of count variation and compositional constraints can have substantial impact on conclusions in the analysis of sequence count data (McMurdie and Holmes, 2014; Silverman et al., 2020; Gloor et al., 2017). Second, Peptostreptococcaceae has been found to be decreased in CD based on the analysis of independent data (Imhann et al., 2018). Third, in visualizing the count data for Peptostreptococcaceae and Veillonellaceae (Figure S8) we find no visual difference in Veillonellaceae but a notable difference in Peptostreptococcaceae. Therefore, we conclude that our approach has revealed novel associations in this data and excluded potentially spurious conclusions.

## 7. Inferring Microbial Trajectories in an Artificial Gut Model

Artificial gut models provide a powerful *in vitro* approach to studying microbial communities. To demonstrate the generality of our inference methods for Marginally LTP models, we reanalyzed a previously published high-resolution longitudinal study of 4 artificial gut models using a GMGP model (Silverman et al., 2018a). Each of the 4 artificial gut models represent a closed system that were maintained in nearly identical conditions and inoculated with an identical fecal slurry. Following Silverman et al. (2018a), We therefore chose to model each of the four vessels (r∈1,…,4) as independent but with a shared covariance structure using the following GMGP model:

$$Y_{\cdot \text{tr}}\sim \text{Multinomial}\left(n_{\text{tr}},\pi_{\cdot \text{tr}}\right)$$

$$\pi_{\cdot \text{tr}}={\text{ALR}}_{D}^{-1}\left(\eta_{\cdot \text{tr}}\right)$$

$$\eta_{\cdot \text{tr}}\sim N(\Lambda ({X.}_{\text{tr}}),\Sigma (Z))$$

$$\Lambda \sim \text{GP}\left(\Theta ,\Sigma ,\Gamma_{\text{vessel}}\circ \Gamma_{\text{time}}\right)$$

Σ~IWP(Ξ,ν)

where Ξ is a kernel based on sequence similarity between bacterial taxa, $\Gamma_{\text{time}}$ is a squared exponential kernel based on the time between samples, $\Gamma_{\text{vessel}}$ is a block identity kernel, and ∘ denote the element-wise multiplication of kernel functions. To evaluate the impact of using small values for the degree-of-freedom parameter ν in model priors, we set ν=D+2. Details on these kernels as well as the matrix functions Θ are described further in Appendix J. The above GMGP model was inferred using the function *basset* from the R package *fido*. While there are differences between the generalized dynamic linear model used in Silverman et al. (2018a) and the above GMGP model, it is notable that the model used in Silverman et al. (2018a) took on the order of 5 hours while the GMGP model above, using CU sampler with Laplace approximation, ran in just 4 seconds.

Our results are in general agreement with those of Silverman et al. (2018a). Notably, we found a distinct decrease in the relative amount of the family Bacteroidaceae immediately after the introduction of a B. *ovatus* probiotic at hour 60. Still, our analyses revealed a number of features unappreciated in prior analyses. Most notably, our GMGP analyses suggests that the degree to which the community was undergoing sub-daily oscillations was far greater than was appreciated in Silverman et al. (2018a). Silverman et al. (2018a) noticed that the relative amount of Enterobacteriaceae displayed distinct, unsyncronized, sub-daily oscillations in all four artificial gut vessels. We too found evidence of unsyncronized sub-daily oscillations in all four vessels. However, we also found such oscillatory dynamics in numerous other dimensions including the CLR coordinates corresponding to the Lachnospiraceae, Desulfovibrionaceae, and Synergistaceae. We suspect that the flexibility provided by the non-linear GMGP model allowed these oscillatory patterns to be more easily revealed than the random walk dynamics originally modeled in Silverman et al. (2018a).

## 8. Conclusion

In this work we have developed efficient inference for the analysis of a large class of multinomial logistic-normal models through the use of a shared marginal representation. We demonstrated that, in comparison to HMC, the CU sampler with a marginal Laplace approximation improved sampler efficiency by up to 5 orders of magnitude while preserving accuracy of point estimation and uncertainty quantification. Yet, the performance of our Laplace approximation under observation distributions beyond the log-ratio parameterized multinomial is more uncertain. We hypothesize that our results could generalize to other exponential family distributions parameterized by natural parameters since such distributions are globally log-concave. Yet, we expect that there are other observation distributions where a Laplace approximation to the LTP form may be sub-optimal. Rather than resorting to using MCMC to infer the collapsed model form, we suggest that methods of particle refinement of the initial Laplace approximation (*e.g*., parallel MCMC steps for each sample from the LTP form, or sequential importance resampling) may be more efficient. We believe such extensions are prime areas for future work.

Here we have compared the CU sampler with marginal Laplace approximation against HMC and VB for inference of MLN models, yet many other comparisons are possible. In particular, both the Integrated Nested Laplace Approximation (INLA) (Rue et al., 2009) and Pólya-gamma data-augmentation (Polson et al., 2013) are popular approaches for inferring Bayesian logistic models. Like INLA, our approach uses Laplace approximations to posterior marginals; yet INLA’s requires that the number of hyper-parameters is small (e.g., ≤ 6) which proves limiting in inferring MLN models with potentially dense co variation between multinomial categories as we address here. In contrast to INLA, Pólya-gamma data augmentation uses a Gibbs sampling algorithm with augmented Pólya-gamma random variables. Yet numerous authors have found that Pólya-gamma data augmentation is too slow for scalable inference of MLN models due to two key limitations (Linderman et al., 2015; Glynn et al., 2019; Zhang and Zhou, 2017). First, MLN models do not permit block updates to Pólya-gamma random variables and as a result, the number of Gibbs steps required for each posterior sample scales linearly with the number of multinomial categories (Polson et al., 2013; Linderman et al., 2015; Zhang and Zhou, 2017). Second, when the number of multinomial categories is large, sampling Pólya-gamma random variables can become rate limiting (Glynn et al., 2019). Rather than INLA or Pólya-gamma data augmentation, we believe that the most fruitful comparisons involve alternative approximations for sampling the collapsed representation of Marginally LTP models. Notably, for inference of hierarchical Bayesian Gaussian processes, multiple authors have found expectation propagation to be more accurate, albeit an order of magnitude slower than, Laplace approximation (Jylänki et al., 2011; Nickisch and Rasmussen, 2008; Kuss et al., 2005). Overall, further comparisons will both help to clarify the use cases for the CU sampler with marginal Laplace approximation and point to potential future improvements.

One limitation of this work is that our derived error bound required the assumption that ν→∞. This assumption was required so that the Matrix-t distribution was globally log-convex – a requirement of the theory introduced in (Ogden, 2018). In practice however, we expect practitioners to use finite values of ν and in these cases our error bound serves only as a tool for building intuition regarding the error rate of our Laplace approximation. Despite this limitation, our analyses of both simulated and real data suggest that the Laplace approximation provides accurate inference even when ν is small. Still, we expect some degradation of the accuracy of the Laplace approximation for smaller values of ν compared to larger values of ν.

## Supplementary Material

Supplement

1

## Figures and Tables

**Figure 3:**
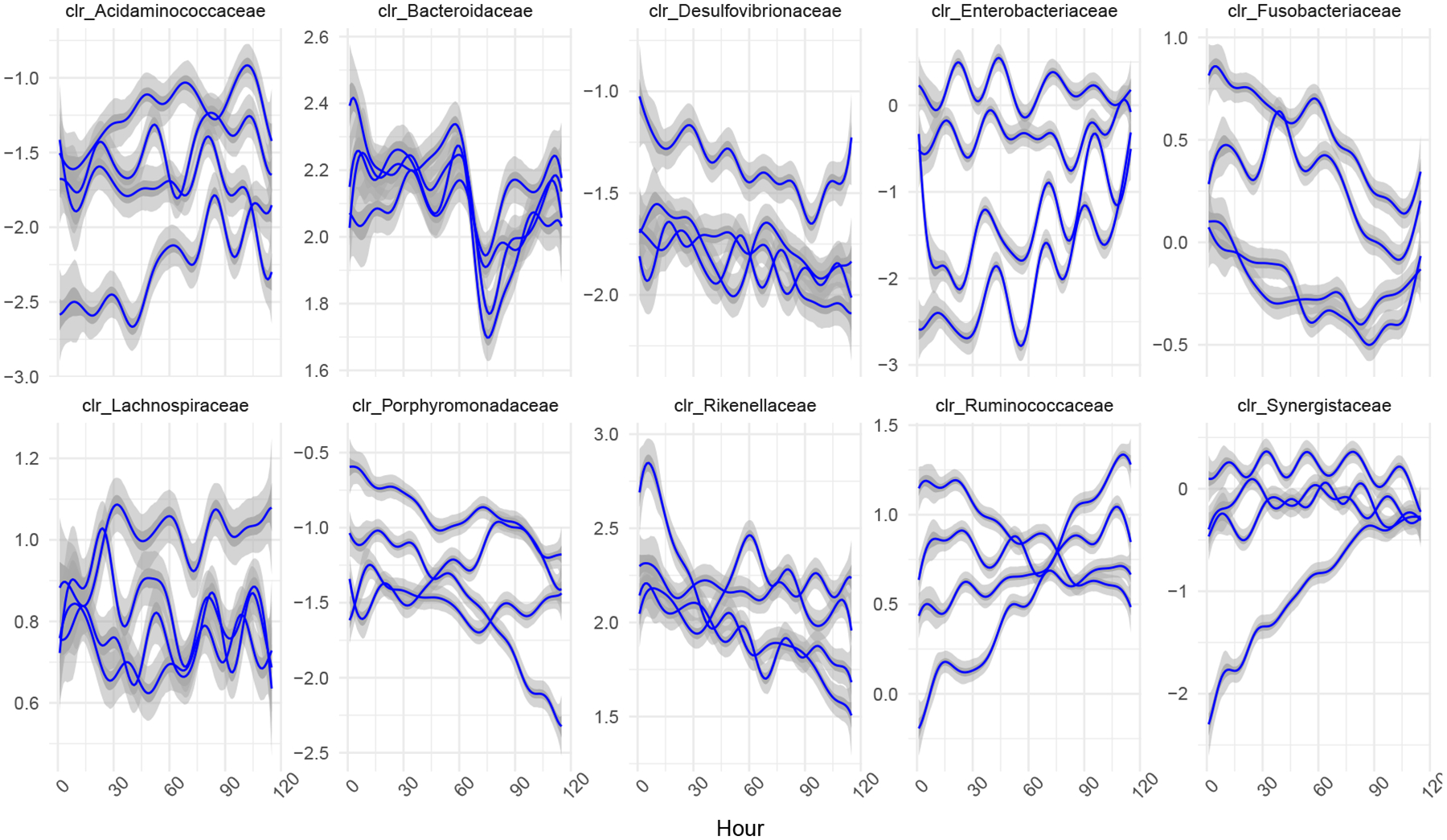
Posterior mean and credible intervals for Λ of *fido*::*basset* applied to artificial gut data. Following Silverman et al. (2018a) we analyze the data from 4 independent artificial guts at the bacterial family level. The posterior mean as well as 50% and 95% credible regions for Λ are depicted. The posterior is depicted with respect to centered log-ratio (CLR) coordinates so that each coordinate could be identified with a different bacterial family.

## References

[R1] Äijö Tarmo, Müller Christian L, and Bonneau Richard. Temporal probabilistic modeling of bacterial compositions derived from 16s rRNA sequencing. Bioinformatics, 34(3):372380, 2017. 10.1093/bioinformatics/btx549 PMC5860357 28968799

[R2] Aitchison J. The statistical analysis of compositional data. Monographs on statistics and applied probability. Chapman and Hall, London; New York, 1986. ISBN 0412280604 (U.S.).

[R3] Aitchison J and Shen SM. Logistic-normal distributions - some properties and uses. Biometrika, 67(2):261–272, 1980. ISSN 0006-3444. doi: Doi 10.2307/2335470.

[R4] Billheimer Dean, Guttorp Peter, and Fagan William F. Statistical interpretation of species composition. Journal of the American Statistical Association, 96(456):1205–1214, 2001.

[R5] Blei David M and Lafferty John D. Dynamic topic models. In Proceedings of the 23rd international conference on Machine learning, pages 113–120. ACM, 2006.

[R6] Cargnoni Claudia, Müller Peter, and West Mike. Bayesian forecasting of multinomial time series through conditionally Gaussian dynamic models. Journal of the American Statistical Association, 92(438):640–647, 1997. ISSN 0162-1459.

[R7] Gelman Andrew, Lee Daniel, and Guo Jiqiang. Stan: A probabilistic programming language for Bayesian inference and optimization. Journal of Educational and Behavioral Statistics, 40(5):530–543, 2015. ISSN 1076-9986.

[R8] Gevers Dirk, Kugathasan Subra, Denson Lee A, Vázquez-Baeza Yoshiki, Van Treuren Will, Ren Boyu, Schwager Emma, Knights Dan, Song Se Jin, Yassour Moran, The treatment-naive microbiome in new-onset Crohn’s disease. Cell host & microbe, 15(3): 382–392, 2014. 24629344 10.1016/j.chom.2014.02.005 PMC4059512

[R9] Gloor Gregory B, Macklaim Jean M, Pawlowsky-Glahn Vera, and Egozcue Juan J. Microbiome datasets are compositional: and this is not optional. Frontiers in Microbiology, 8: 2224, 2017. 29187837 10.3389/fmicb.2017.02224 PMC5695134

[R10] Gloor Gregory Brian, Macklaim Jean M., Vu Michael, and Fernandes Andrew D.. Compositional uncertainty should not be ignored in high-throughput sequencing data analysis. Austrian Journal of Statistics, 45(4):73, 2016. doi: 10.17713/ajs.v45i4.122.

[R11] Glynn Chris, Tokdar Surya T, Howard Brian, Banks David L, Bayesian analysis of dynamic linear topic models. Bayesian Analysis, 14(1):53–80, 2019.

[R12] Grantham Neal S., Reich Brian J., Borer Elizabeth T., and Gross Kevin. Mimix: a Bayesian mixed-effects model for microbiome data from designed experiments. ArXiv e-prints, 1703:arXiv:1703.07747, 2017.

[R13] Gupta Arjun K and Nagar Daya K. Matrix variate distributions. Chapman and Hall/CRC, 2018.

[R14] Imhann Floris, Vila Arnau Vich, Bonder Marc Jan, Fu Jingyuan, Gevers Dirk, Visschedijk Marijn C, Spekhorst Lieke M, Alberts Rudi, Franke Lude, Van Dullemen Hendrik M, Interplay of host genetics and gut microbiota underlying the onset and clinical presentation of inflammatory bowel disease. Gut, 67(1):108–119, 2018. 27802154 10.1136/gutjnl-2016-312135 PMC5699972

[R15] Jordan Michael. The exponential family: Basics, 2010. URL https://people.eecs.berkeley.edu/~jordan/courses/260-spring10/other-readings/chapter8.pdf.

[R16] Jostins Luke, Ripke Stephan, Weersma Rinse K, Duerr Richard H, McGovern Dermot P, Hui Ken Y, Lee James C, Schumm L Philip, Sharma Yashoda, Anderson Carl A, Host-microbe interactions have shaped the genetic architecture of inflammatory bowel disease. Nature, 491(7422):119, 2012. 23128233 10.1038/nature11582 PMC3491803

[R17] Jylänki Pasi, Vanhatalo Jarno, and Vehtari Aki. Robust Gaussian process regression with a Student-t likelihood. Journal of Machine Learning Research, 12(Nov):3227–3257, 2011.

[R18] Kass Robert E and Steffey Duane. Approximate Bayesian inference in conditionally independent hierarchical models (parametric empirical Bayes models). Journal of the American Statistical Association, 84(407):717–726, 1989.

[R19] Khor Chiea Chuen and Hibberd Martin L. Host-pathogen interactions revealed by human genome-wide surveys. Trends in Genetics, 28(5):233–243, 2012. 22445588 10.1016/j.tig.2012.02.001

[R20] Kostic Aleksandar D, Gevers Dirk, Pedamallu Chandra Sekhar, Michaud Monia, Duke Fujiko, Earl Ashlee M, Ojesina Akinyemi I, Jung Joonil, Bass Adam J, Tabernero Josep, Genomic analysis identifies association of Fusobacterium with colorectal carcinoma. Genome research, 22(2):292–298, 2012. 22009990 10.1101/gr.126573.111 PMC3266036

[R21] Kucukelbir Alp, Ranganath Rajesh, Gelman Andrew, and Blei David. Automatic variational inference in Stan. In Advances in Neural Information Processing Systems, pages 568–576, 2015.

[R22] Kuss Malte, Rasmussen Carl Edward, and Herbrich Ralf. Assessing approximate inference for binary Gaussian process classification. Journal of machine learning research, 6(10), 2005.

[R23] Linderman Scott, Johnson Matthew, and Adams Ryan P. Dependent multinomial models made easy: Stick-breaking with the pólya-gamma augmentation. In Advances in Neural Information Processing Systems, pages 3456–3464, 2015.

[R24] MacKay David JC. Choice of basis for Laplace approximation. Machine learning, 33(1): 77–86, 1998.

[R25] McMurdie PJ and Holmes S. Waste not, want not: why rarefying microbiome data is inadmissible. PLoS Computational Biology, 10(4):e1003531, 2014. ISSN 1553-7358 (Electronic) 1553-734X (Linking). doi: 10.1371/journal.pcbi.1003531. 24699258 PMC3974642

[R26] Minka Thomas P. Old and new matrix algebra useful for statistics, 2000. URL www.stat.cmu.edu/minka/papers/matrix.html.

[R27] Nickisch Hannes and Rasmussen Carl Edward. Approximations for binary Gaussian process classification. Journal of Machine Learning Research, 9(Oct):2035–2078, 2008.

[R28] Ogden Helen. On the error in Laplace approximations of high-dimensional integrals. arXiv:1808.06341, 2018.

[R29] Pawlowsky-Glahn Vera, Egozcue Juan José, and Tolosana-Delgado Raimon. Modeling and analysis of compositional data. John Wiley & Sons, 2015.

[R30] Polson Nicholas G, Scott James G, and Windle Jesse. Bayesian inference for logistic models using pólya-gamma latent variables. Journal of the American statistical Association, 108 (504):1339–1349, 2013.

[R31] Prado Raquel and West Mike. Time series: modeling, computation, and inference. Chapman & Hall/CRC texts in statistical science series. CRC Press, Boca Raton, 2010. ISBN 9781420093360.

[R32] Quintana Jose M and West Mike. An analysis of international exchange rates using multivariate DLMs. The Statistician, pages 275–281, 1987.

[R33] Rasmussen Carl Edward. Gaussian processes in machine learning. In Summer School on Machine Learning, pages 63–71. Springer, 2003.

[R34] Riihimäki Jaakko, Vehtari Aki, Laplace approximation for logistic Gaussian process density estimation and regression. Bayesian analysis, 9(2):425–448, 2014.

[R35] Rossi PE, Allenby GM, and McCulloch R. Bayesian Statistics and Marketing. Wiley Series in Probability and Statistics. Wiley, 2012. ISBN 9780470863688.

[R36] Rue Håvard, Martino Sara, and Chopin Nicolas. Approximate Bayesian inference for latent Gaussian models by using integrated nested Laplace approximations. Journal of the royal statistical society: Series b (statistical methodology), 71(2):319–392, 2009.

[R37] Shah Amar, Wilson Andrew, and Ghahramani Zoubin. Student-t processes as alternatives to Gaussian processes. In Artificial Intelligence and Statistics, pages 877–885, 2014.

[R38] Silverman JD, Washburne AD, Mukherjee S, and David LA. A phylogenetic transform enhances analysis of compositional microbiota data. eLife, 6, 2017. ISSN 2050-084X (Electronic) 2050-084X (Linking). doi: 10.7554/eLife.21887. PMC5328592 28198697

[R39] Silverman JD, Durand HK, Bloom RJ, Mukherjee S, and David LA. Dynamic linear models guide design and analysis of microbiota studies within artificial human guts. Microbiome, 6(1):202, 2018a. ISSN 2049-2618 (Electronic) 2049-2618 (Linking). doi: 10.1186/s40168-018-0584-3. 30419949 PMC6233358

[R40] Silverman Justin D. fido: Multinomial logistic normal models, 2019. URL https://github.com/jsilve24/fido.

[R41] Silverman Justin D, Roche Kimberly, Mukherjee Sayan, and David Lawrence A. Naught all zeros in sequence count data are the same. Computational and Structural Biotechnology Journal, 18:2789, 2020. 33101615 10.1016/j.csbj.2020.09.014 PMC7568192

[R42] Stan Development Team. Stan user’s guide, 2018. URL https://mc-stan.org/docs/2_18/stan-users-guide/index.html.

[R43] Strauss Jaclyn, Kaplan Gilaad G, Beck Paul L, Rioux Kevin, Panaccione Remo, DeVinney Rebekah, Lynch Tarah, and Allen-Vercoe Emma. Invasive potential of gut mucosaderived Fusobacterium nucleatum positively correlates with IBD status of the host. Inflammatory Bowel Diseases, 17(9):1971–1978, 2011. 21830275 10.1002/ibd.21606

[R44] Sun Shiliang, Cao Zehui, Zhu Han, and Zhao Jing. A survey of optimization methods from a machine learning perspective. IEEE Transactions on Cybernetics, 2019. 10.1109/TCYB.2019.2950779 31751262

[R45] West Mike and Harrison Jeff. Bayesian forecasting and dynamic models. Springer series in statistics. Springer, New York, 2nd edition, 1997. ISBN 0387947256.

[R46] Zhang Quan and Zhou Mingyuan. Permuted and augmented stick-breaking Bayesian multinomial regression. The Journal of Machine Learning Research, 18(1):7479–7511, 2017.

[R47] Álvarez Mauricio A., Rosasco Lorenzo, and Lawrence Neil D.. Kernels for vector-valued functions: A review. Foundations and Trends in Machine Learning, 4(3):195, 00 2012. doi: 10.1561/2200000036.

