# Supplementary material for: Bayesian Multinomial Logistic Normal Models through Marginally Latent Matrix-T Processes
Source: J Mach Learn Res. Author manuscript; Available in PMC 2026 Apr 9. (PMC13061366)
Supplement: Supplement [file NIHMS2137087-supplement-Supplement.pdf]

Bayesian Multinomial Logistic Normal Models through Marginally Latent Matrix-T Processes: Supplementary Figures

Justin D. Silverman[1,2,3,4], Kimberly Roche[5], Zachary C. Holmes[6], Lawrence A. David[5,6,8,9], and Sayan Mukherjee[5,7,8,9]

[1]*College of Information Science and Technology, Penn State University, University Park, PA 16802*
[2]*Department of Statistics, Penn State University, University Park, PA 16802*
[3]*Institute for Computational and Data Science, Penn State University, University Park, PA 16802*
[4]*Department of Medicine, Penn State University, Hershey, PA 17033*
[5]*Program in Computational Biology and Bioinformatics, Duke University, Durham, NC 27708*
[6]*Department of Molecular Genetics and Microbiology, Duke University, Durham, NC 27708*
[7]*Departments of Statistical Science, Mathematics, Computer Science, Biostatistics & Bioinformatics, Duke University, Durham, NC 27708*
[8]*Center for Genomic and Computational Biology, Duke University, Durham, NC 27708*
[9]*Denotes Co-corresponding Authors*

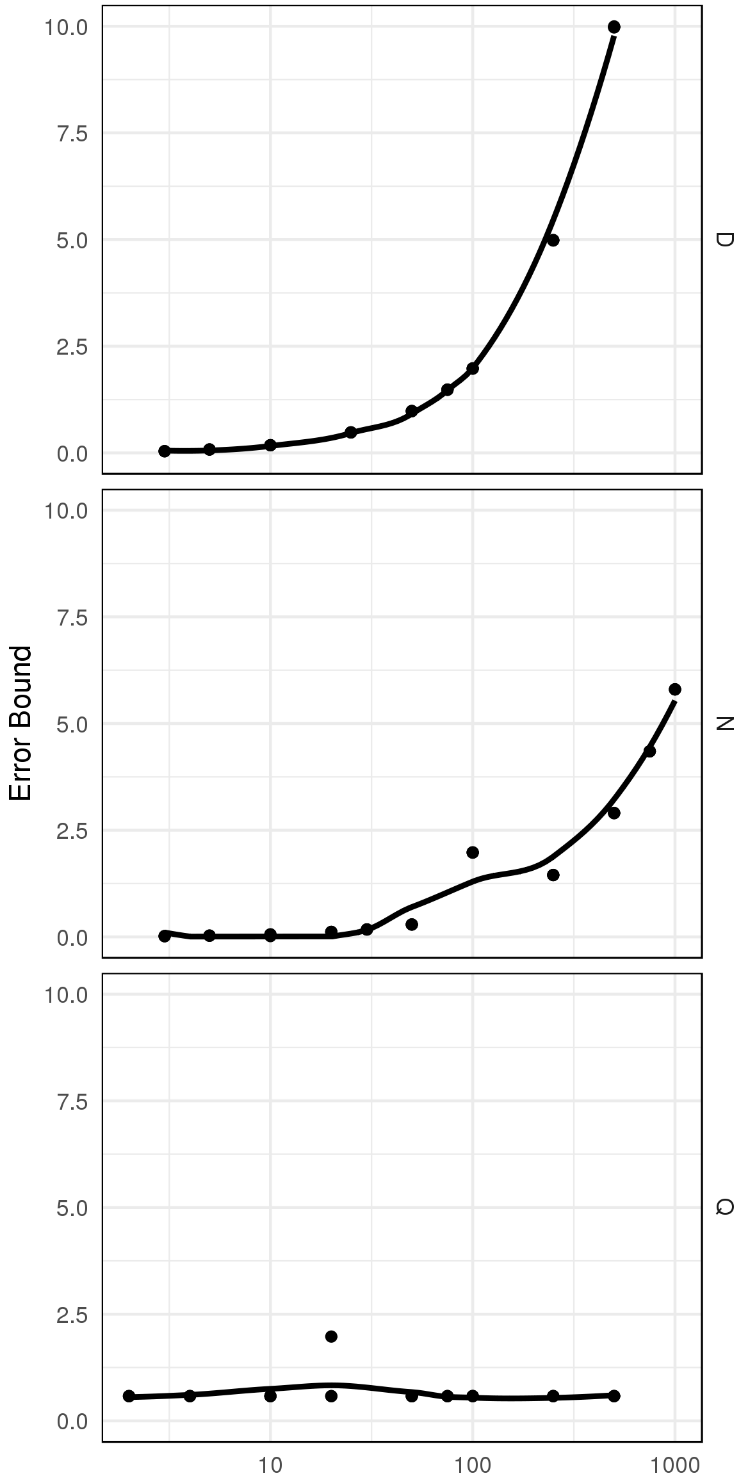

Figure S1: **The Error bound, which was described in the main text, evaluated for each simulation.**

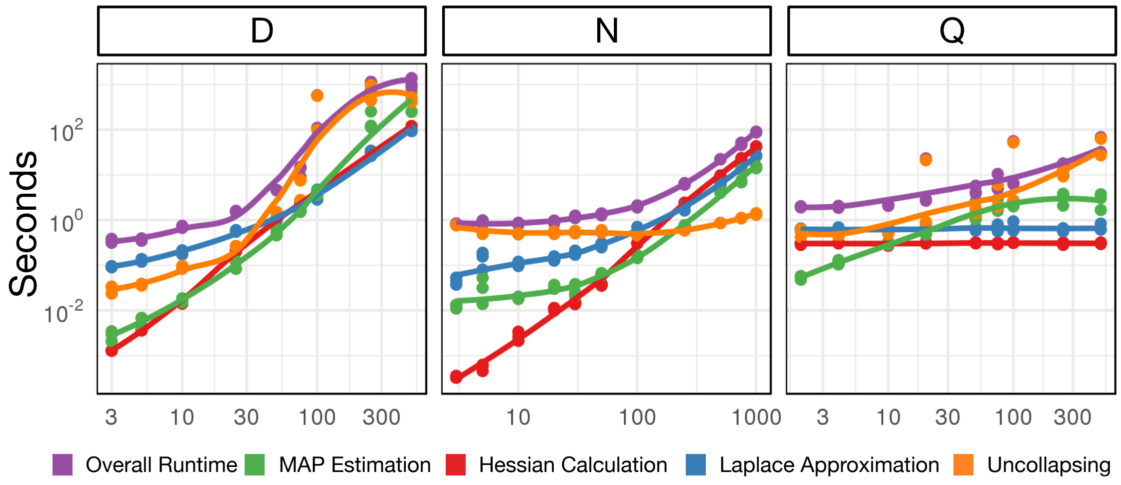

Figure S2: **LA Collapsed run-times decomposed into component processes.**

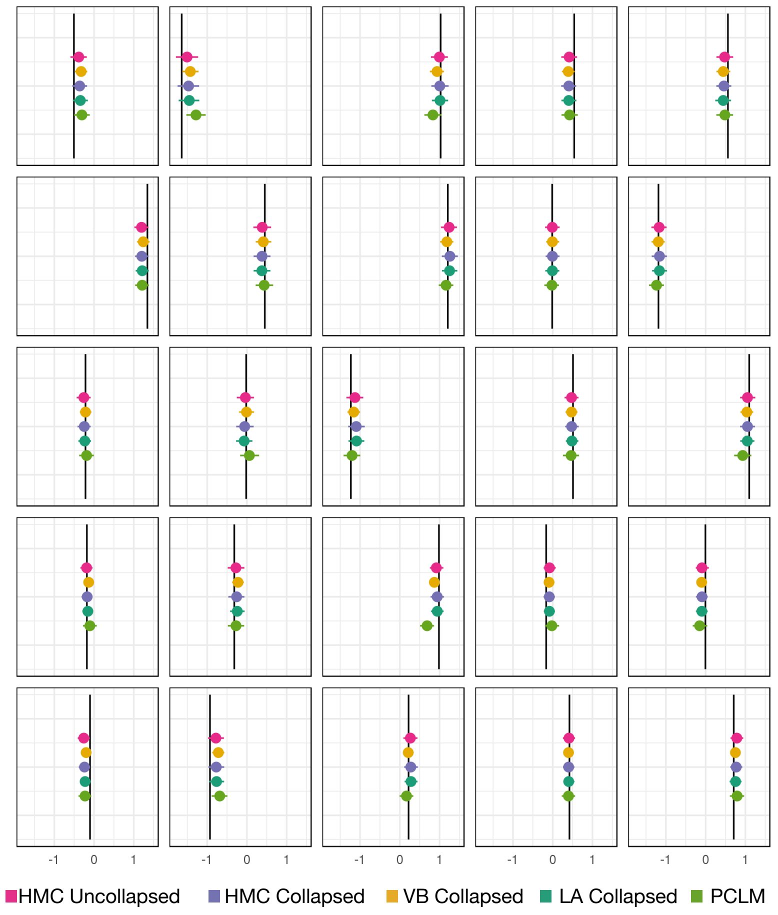

Figure S3: **Example of simulation in which uncertainty quantification from LA Collapsed agrees with estimates from HMC.** For this simulation $N = 30$, $D = 30$, $Q = 5$. Each panel represents a different element Λ_{ij}. The true simulated value of Λ_{ij} in each panel is denoted by a black vertical line.

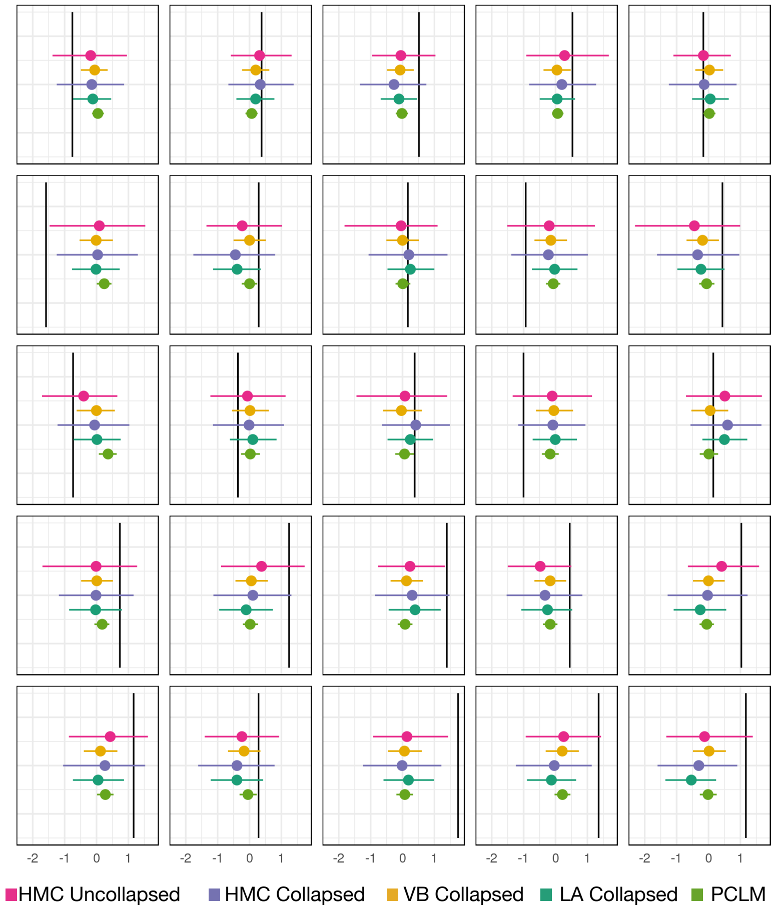

Figure S4: **Example of simulation in which uncertainty quantification from LA Collapsed disagrees with estimates from HMC.** For this simulation $N = 100$, $D = 30$, $Q = 250$. Each panel represents a different element Λ_{ij}. The true simulated value of Λ_{ij} in each panel is denoted by a black vertical line.

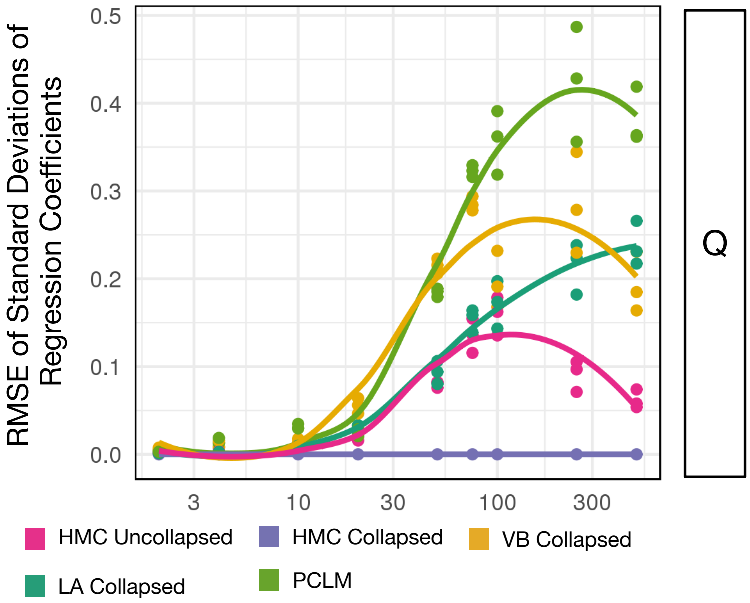

Figure S5: **Uncertainty quantification error can be contextualized by comparing against a fifth implementation (PCLM)**. The PCLM model consists of a pseudo-count based estimate of η followed by the direct application of a multivariate conjugate linear model to estimate parameters Λ and Σ. In this way the PCLM model ignores multinomial variation.

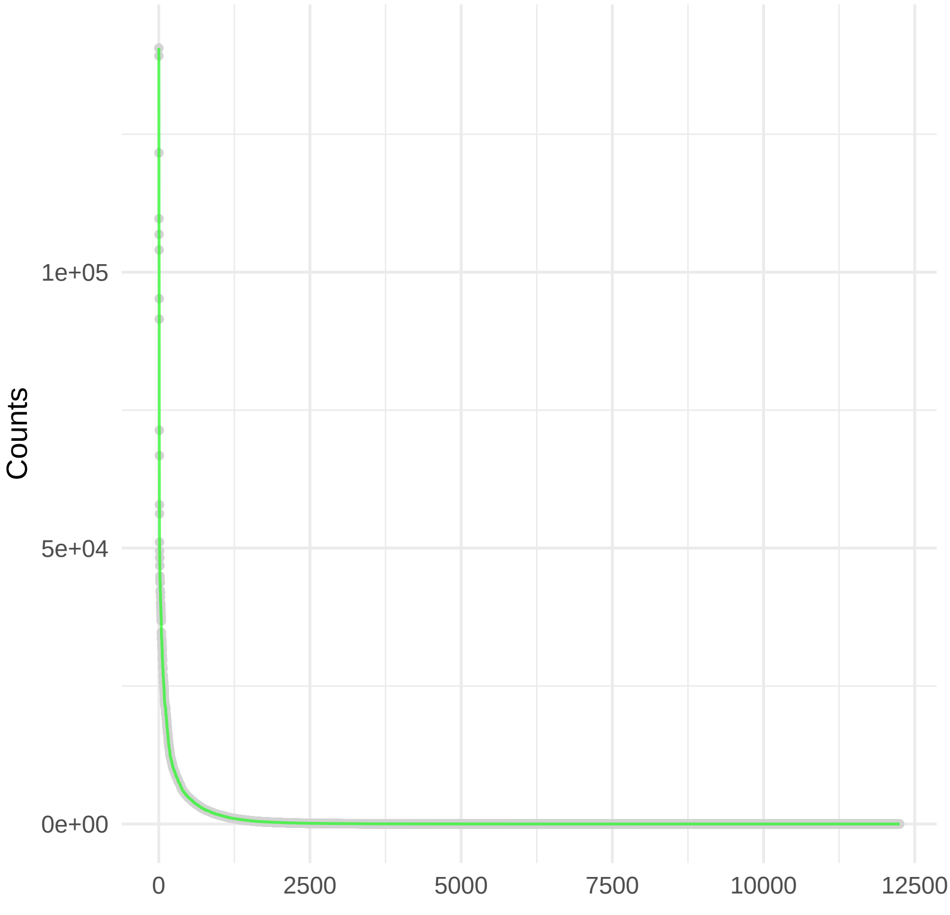

Figure S6: **Posterior predictive checks from *i.e.,* LA Collapsed applied to real data.** Each element of data Y is ordered by value and denoted by a green line. The marginal posterior predictive distribution of each element is displayed based on its mean and 95% credible interval in Grey.

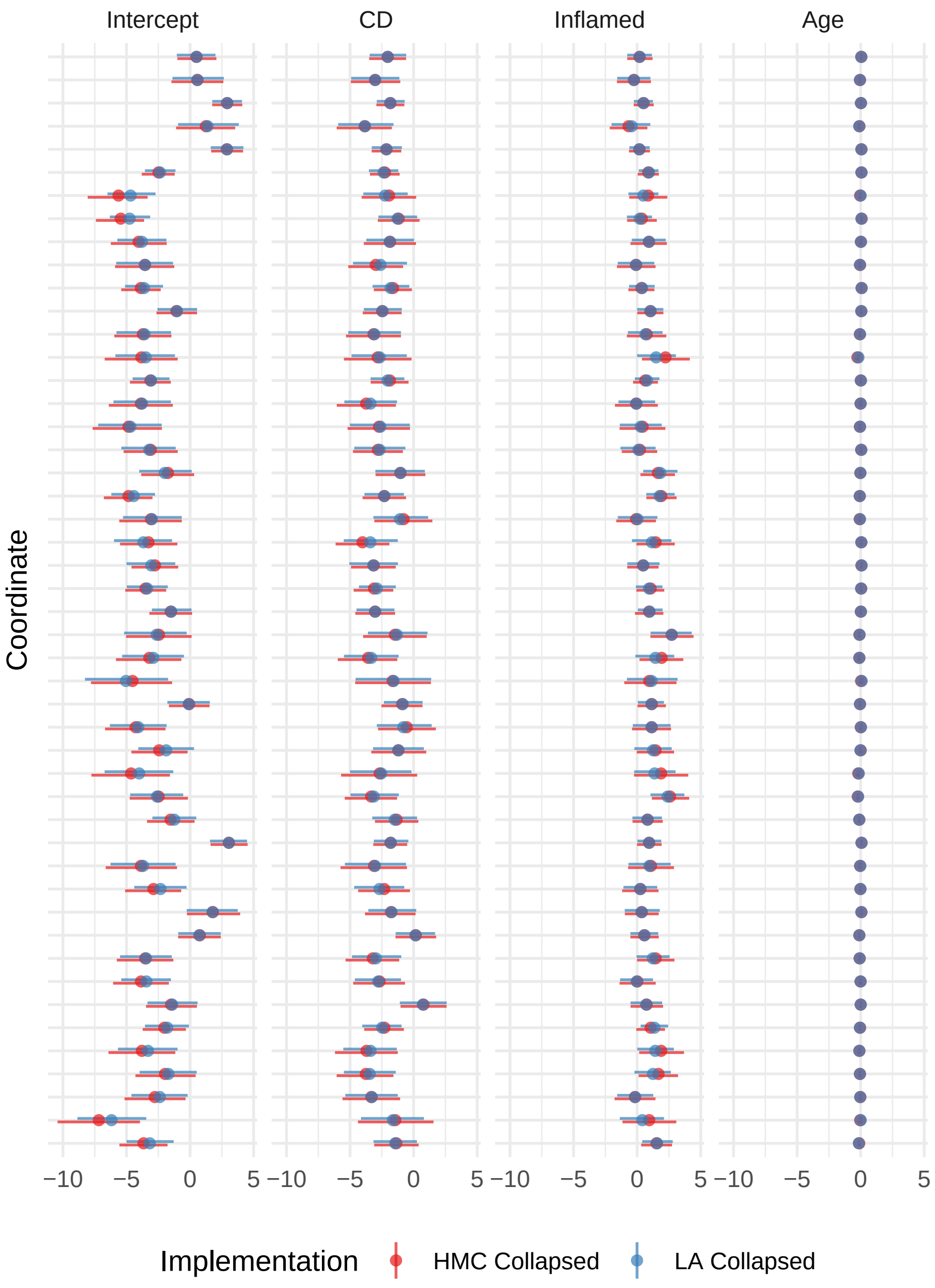

Figure S7: **Posterior estimates of Λ produced by HMC Collapsed and LA Collapsed are similar.** For each implementation the Posterior mean and 95% credible interval is indicated.

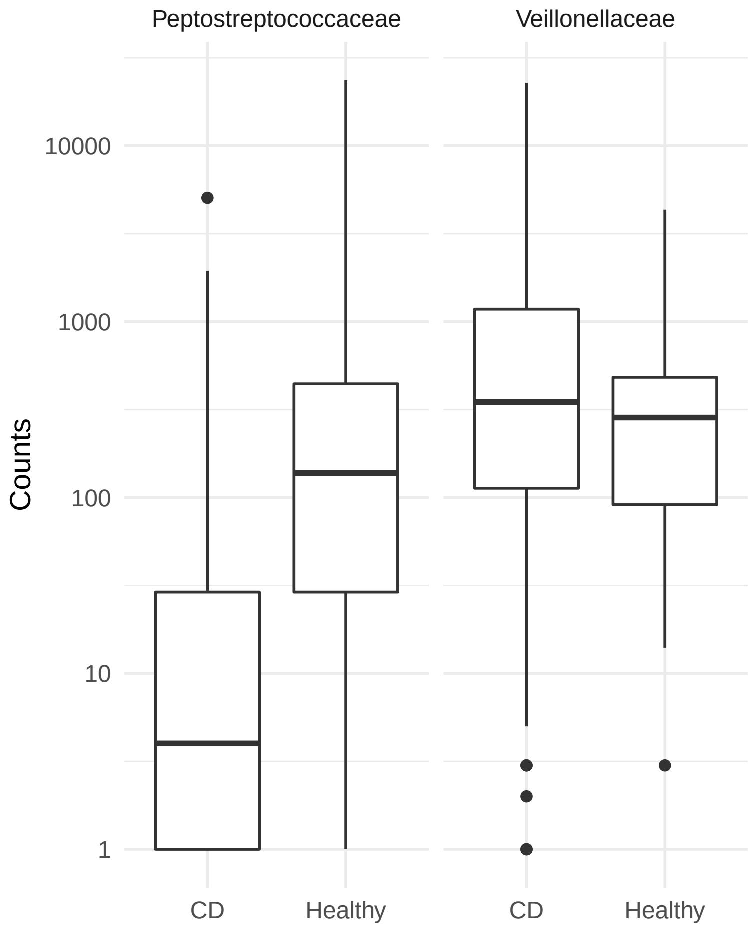

Figure S8: **Comparison of raw counts for CD and Healthy groups for Peptostreptococcaceae and Veillonellaceae families from Real Data analysis.** To allow visualization, a pseudo-count of 1 was added prior to log-transformation to avoid taking the log of zero. Boxplots show median, IQR, and 1.5×IQR.
