# Supplementary material for: Bayesian Multinomial Logistic Normal Models through Marginally Latent Matrix-T Processes
Source: J Mach Learn Res. Author manuscript; Available in PMC 2026 Apr 9. (PMC13061366)
Supplement: 1 [file NIHMS2137087-supplement-1.pdf]

Appendix A. Generalized Multivariate Conjugate Linear (GMCL) Models

Here we prove that the GMCL models defined in Equations (6)-(10) are Marginally LTP models, and in so doing, derive their collapsed (LTP) form. Additionally, we demonstrate that uncollapsing the LTP form can be done efficiently. Our proof relies on the following affine transformation property of the matrix normal distribution. Given matrices A, B, and C, as well as a random matrix $X \sim N(M, U, V)$; then for a random matrix $Z = A + BXC$ we have $Z \sim N(A + BMC, BUB^T, C^TVC)$ (Gupta and Nagar, 2018, p. 64).

Proposition 3 *The GMCL Models, as defined in Equations* (6)-(10)*, are a type of Marginally LTP models.*

Proof We prove this proposition by showing that the marginal $p(\eta, Y, X)$ of GMCL models is an LTP. By Definition 2, if $p(\eta, Y, X)$ is an LTP, then $p(\eta, \Lambda, \Sigma, Y, X)$ is a Marginally LTP model.

To begin, we note that equations (8)-(10) can alternatively be written as

$$\eta = \Lambda X + E^\eta \quad E^\eta \sim N(0, \Sigma, I_N) \tag{26}$$

$$\Lambda = \Theta + E^\Lambda \quad E^\Lambda \sim N(0, \Sigma, \Gamma) \tag{27}$$

$$\Sigma \sim IW(\Xi, \upsilon). \tag{28}$$

Using this form, in combination with the affine transformation property of the matrix normal distribution stated above, it is straightforward to marginalize over Λ producing the following form:

$$\begin{aligned} \eta &= \Theta X + E^\Lambda X + E^\eta \quad E^\eta \sim N(0, \Sigma, I_N) \quad E^\Lambda \sim N(0, \Sigma, \Gamma) \\ &= \Theta X + E^* \quad E^\star \sim N(0, \Sigma, I_N + X^T \Gamma X). \end{aligned} \tag{29}$$

Thus we may rewrite Equations (26)-(28) as

$$\eta = \Theta X + E^* \quad E^\star \sim N(0, \Sigma, I_N + X^T \Gamma X) \tag{30}$$

$$\Sigma \sim IW(\Xi, \upsilon). \tag{31}$$

By using the definition of the matrix-t given in Section 3.1 we can marginalize over Σ in Equations (30) and (31) to get

$$\eta \sim T(\upsilon, \Theta X, \Xi, I_N + X^T \Gamma X).$$

Finally, incorporating equations (6) and (7) allows us to write the marginalized form of GMCL models, $p(\eta, Y, X)$, as an LTP

$$\begin{aligned} Y &\sim f(\pi) \\ \pi &= \phi^{-1}(\eta) \\ \eta &\sim T(\upsilon, B, K, A) \end{aligned}$$

where $B = \Theta X$, $K = \Xi$, and $A = I_N + X^T \Gamma X$. ∎

Next, we demonstrate that for GMCL models, the conditional posterior $p(\Lambda, \Sigma|\eta, Y, X)$ can be computed and sampled efficiently: That the collapsed model can be uncollapsed efficiently. As Λ and Σ are conditionally independent of Y given η in GMCL models, we may write

$$p(\Lambda, \Sigma \mid \eta, Y, X) = p(\Lambda, \Sigma \mid \eta, X) = p(\Lambda \mid \Sigma, \eta, X)\, p(\Sigma \mid \eta, X).$$

The right hand side of the above equation represents the posterior of a multivariate conjugate linear model that can be sampled efficiently using the following relations (Rossi et al., 2012, p. 32):

$$\begin{aligned}
\upsilon_N &= \upsilon + N \\
\Gamma_N &= (XX^T + \Gamma^{-1})^{-1} \\
\Lambda_N &= (\eta X^T + \Theta\Gamma^{-1})\Gamma_N \\
\Xi_N &= \Xi + (\eta - \Lambda_N X)(\eta - \Lambda_N X)^T + (\Lambda_N - \Theta)\Gamma^{-1}(\Lambda_N - \Theta)^T \\
p(\Sigma|\eta, X) &= IW(\Xi_N, \upsilon_N) \\
p(\Lambda|\Sigma, \eta, X) &= N(\Lambda_N, \Sigma, \Gamma_N).
\end{aligned}$$

Appendix B. Generalized Multivariate Dynamic Linear Model (GMDLM)

Here we prove that the GMDLMs defined in Equations (11)-(16) are Marginally LTP models. Additionally we provide a recursive procedure for uncollapsing an LTP to a GMDLM.

B.1 Derivation of Collapsed Form

Proposition 4 *The GMDLMs defined in Equations* (11)-(16)*, are a type of Marginally LTP models.*

Proof As in Proposition 3, we show that GMDLMs are Marginally LTPs by showing that a marginal of the GMDLMs, $p(\eta, Y)$, is an LTP.

We begin by deriving the marginal distribution $p(\eta, Y)$ in terms of the quantities F_t, G_t, W_t, Σ, M_0 and C_0. As all densities involved are multivariate or matrix-variate normal, the result must also be multivariate or matrix-variate normal and thus fully described by the mean and covariance of η. To derive the mean and covariance we first derive a useful alternative representation of η_t^T with respect to Θ_{t-k-1} for some positive integer $k < t$.

Substituting Equation (14) into Equation (13) allows η_t^T be expressed with respect to Θ_{t-1} as

$$\eta_t^T = F_t^T G_t \Theta_{t-1} + F_t^T \Omega_t + \nu_t^T. \tag{32}$$

Repeated substitution of Θ_{t-k} leads to the following form for η_t^T in terms of Θ_{t-k-1}

$$\eta_t^T = F_t^T \mathcal{G}_{t:t-k} \Theta_{t-k-1} + F_t^T \Omega_t + \sum_{\ell=t}^{t-k-1} F_t^T \mathcal{G}_{t:\ell} \Omega_{\ell-1} + \nu_t^T \tag{33}$$

where $\mathcal{G}_{t:t-k}$ is shorthand for $G_t G_{t-1} \cdots G_{t-k}$. Using the affine transformation property of the matrix normal given in Appendix A in combination with (15) we can marginalize over the random variables $\Omega_t, \ldots, \Omega_1, \nu_t$ in Equation (33) giving

$$\eta_t^T \sim N\left(F_t^T \mathcal{G}_{t:1} M_0, \gamma_t + F_t^T \left[W_t + \sum_{\ell=t}^{2} \mathcal{G}_{t:\ell} W_{\ell-1} \mathcal{G}_{\ell:t}^T + \mathcal{G}_{t:1} C_0 \mathcal{G}_{1:t}^T\right] F_t, \Sigma\right). \quad (34)$$

Next we calculate $Cov(\eta_t^T, \eta_{t-k}^T)$. In parallel to Equation (32) we may write η_{t-k}^T as

$$\eta_{t-k}^T = F_{t-k}^T G_{t-k} \Theta_{t-k-1} + F_{t-k}^T \Omega_{t-k} + \nu_{t-k}^T. \quad (35)$$

Using Equation (33) and (35) along with the fact $Cov(AX_1 + BX_2, Y) = A\,Cov(X_1, Y) + B\,Cov(X_2, Y)$ and that $Cov(\Theta_s, \nu_\ell) = Cov(\Theta_s, \Omega_\ell) = Cov(\Omega_\ell, \nu_s) = 0$ for all s and ℓ, we can write

$$Cov(\eta_t^T, \eta_{t-k}^T) = F_t^T \mathcal{G}_{t:t-k} Var(\theta_{t-k-1}) G_{t-k}^T F_{t-k} + F_t^T \mathcal{G}_{t:t-k+1} Var(\Omega_{t-k}) F_{t-k} \quad (36)$$

where $Var(\Theta_{t-k-1})$ can be written recursively as

$$Var(\Theta_{t-k-1}) = G_{t-k-1} Var(\Theta_{t-k-2}) G_{t-k-1}^T + Var(\Omega_{t-k-1})$$

and where $Var(\Omega_{t-k-1}) = \Sigma \otimes W_{t-k-1}$. Combining this recursive form with equation (36) gives

$$\begin{aligned} Cov(\eta_t^T, \eta_{t-k}^T) = F_t^T \mathcal{G}_{t:t-k+1}(\Sigma \otimes W_{t-k}) F_{t-k} + \sum_{\ell=t-k}^{2} F_t^T \mathcal{G}_{t:\ell}(\Sigma \otimes W_{\ell-1}) G_{\ell:t-k}^T F_{t-k} \\ + F_t^T \mathcal{G}_{t:1}(\Sigma \otimes C_0) G_{1:t-k}^T F_{t-k}. \quad (37) \end{aligned}$$

Together Equations (34) and (37) characterize the marginal distribution of η_t^T in terms of F_t, G_t, W_t, Σ, M_0 and C_0. Noting that if $X \sim N(M, U, V)$ then $X^T \sim N(M^T, V, U)$, it follows that

$$\begin{aligned} \eta &\sim N(B, \Sigma, A) \\ B &= \begin{bmatrix} | & & | & & | \\ \alpha_1 & \cdots & \alpha_t & \cdots & \alpha_T \\ | & & | & & | \end{bmatrix} \\ \alpha_t &= (F_t^T \mathcal{G}_{t:1} M_0)^T \\ A_{t,t-k} &= \begin{cases} \gamma_t + F_t^T \left[W_t + \sum_{\ell=t}^{2} \mathcal{G}_{t:\ell} W_{\ell-1} \mathcal{G}_{\ell:t}^T + \mathcal{G}_{t:1} C_0 \mathcal{G}_{1:t}^T\right] F_t \text{ if } k = 0 \\ F_t^T \left[\mathcal{G}_{t:t-k+1} W_{t-k} + \sum_{\ell=t-k}^{2} \mathcal{G}_{t:\ell} W_{\ell-1} G_{\ell:t-k}^T + \mathcal{G}_{t:1} C_0 G_{1:t-k}^T\right] F_{t-k} \text{ if } k > 0 \end{cases} \end{aligned}$$

Finally, using the marginalization property of the matrix normal and the inverse Wishart used in our definition of the matrix-t distribution and incorporating Equations (11), (12) and (16) it follows that

$$Y \sim f(\pi)$$

$$\pi = \phi^{-1}(\eta)$$
$$\eta \sim T(\upsilon, B, \Xi, A).$$

■

B.2 Efficient Form for Uncollapsing

Here we provide an efficient means of sampling from the conditional density $p(\Theta, \Sigma \mid \eta, Y)$ for the GMDLM. First we recognize that Θ is conditionally independent of Y given η. Therefore, our task simplifies to sampling from $p(\Theta, \Sigma \mid \eta)$. The problem is identical to the standard filtering and simulation smoothing problem solved by West and Harrison (1997, p. 603-604). Again, the problem is defined by the following model (which we will refer to as the MDLM model)

$$\eta_t^T = F_t^T \Theta_t + \nu_t^T, \quad \nu_t \sim N(0, \gamma_t \Sigma) \tag{38}$$
$$\Theta_t = G_t \Theta_{t-1} + \Omega_t, \quad \Omega_t \sim N(0, W_t, \Sigma) \tag{39}$$
$$\Theta_0 \sim N(M_0, C_0, \Sigma) \tag{40}$$
$$\Sigma \sim IW(\Xi, \upsilon). \tag{41}$$

Following West and Harrison (1997), below we restate the filtering and retrospective recursions needed to sample from $p(\Theta, \Sigma \mid \eta)$. Note that all densities in this subsection are conditional on the parameters F_t, G_t, W_t, Σ, M_0 and C_0 but that this dependence has been suppressed for notational simplicity. Let us introduce υ_t and Ξ_t as filtering parameters at step t. Further, we define $\upsilon_0 = \upsilon$ and $\Xi_0 = \Xi$. As a final piece of notation we introduce H_t^T as a shorthand for the set $\{\eta_t^T, \dots, \eta_1^T\}$

B.2.1 Filtering Recursions for MDLM Model

(1) Posterior at $t-1$:

$$p(\Sigma \mid H_{t-1}^T) \sim IW(\Xi_{t-1}, \upsilon_{t-1})$$
$$p(\Theta_{t-1} \mid \Sigma, H_{t-1}^T) \sim N(M_{t-1}, C_{t-1}, \Sigma)$$

(2) Prior at t:

$$A_t = G_t M_{t-1}$$
$$R_t = G_t C_{t-1} G_t^T + W_t$$
$$p(\Sigma \mid H_{t-1}^T) \sim IW(\Xi_{t-1}, \upsilon_{t-1})$$
$$p(\Theta_t \mid \Sigma, H_{t-1}^T) \sim N(A_t, R_t, \Sigma)$$

(3) One-step ahead forecast at t:

$$f_t^T = F_t^T A_t$$
$$q_t = \gamma_t + F_t^T R_t F_t$$

$$
\begin{aligned}
p(\Sigma \mid H_{t-1}^T) &\sim IW(\Xi_{t-1}, \upsilon_{t-1}) \\
p(\eta_t \mid \Sigma, H_{t-1}^T) &\sim N(f_t, q_t\Sigma)
\end{aligned}
$$

(4) Posterior at t:

$$
\begin{aligned}
e_t^T &= \eta_t^T - f_t^T \\
S_t &= \frac{R_t F_t}{q_t} \\
M_t &= A_t + S_t e_t^T \\
C_t &= R_t - q_t S_t S_t^T \\
\upsilon_t &= \upsilon_{t-1} + 1 \\
\Xi_t &= \Xi_{t-1} + \frac{e_t e_t^T}{q_t} \\
p(\Sigma \mid H_{t-1}^T) &\sim IW(\Xi_t, \upsilon_t) \\
p(\Theta_t \mid \Sigma, H_t^T) &\sim N(m_t, C_t, \Sigma)
\end{aligned} \tag{42}
$$

Equation (42) differs slightly from the presentation in West and Harrison (1997) as the parameterization of the inverse-Wishart we employ throughout this paper differs from that source. Throughout this work we use the following parameterization for a random matrix $\Sigma \sim IW(\Xi, \upsilon)$:

$$
p(\Sigma) \propto |\Sigma|^{-(P+\upsilon+1)/2} \exp\Big(-\frac{1}{2}\mathrm{tr}\big(\Xi\Sigma^{-1}\big)\Big).
$$

B.2.2 Simulation Smoothing Recursion

The recursions provided here follow directly from Prado and West (2010, p. 268)
(1) Sample $\Sigma \sim IW(\Xi_T, \upsilon_T)$ and then $\Theta_T \sim N(M_t, C_t, \Sigma)$.
(2) For each time t from $T-1$ to 0, sample $p(\Theta_t|\Theta_{t+1}, H_T^T) \sim N(M_t^*, C_t^*, \Sigma)$ where

$$
\begin{aligned}
Z_t &= C_t G_{t+1}^T R_{t+1}^{-1} \\
M_t^* &= M_t + Z_t(\Theta_{t+1} - a_{t+1}) \\
C_t^* &= C_t - Z_t R_{t+1} Z_t^T.
\end{aligned}
$$

Appendix C. Generalized Multivariate Gaussian Process (GMGP) Models

Here we prove that the GMGP models defined in Equations (17)-(21) are marginally LTP models, and in so doing, derive their collapsed (LTP) form. Additionally, we demonstrate that uncollapsing the LTP form can be done efficiently. Finally, we provide a closed form algorithm for predicting and smoothing using GMGP models.

To facilitate this discussion we must first expand our notation to explicitly denote which quantities are inferred via smoothing versus prediction. In the context of Gaussian Processes, smoothing refers to inferring the value of the latent processes Λ and Σ over the finite set (X^o, Z^o) corresponding to observed data (Y). In contrast, prediction refers to inferring

the value of the same latent processes over finite sets that do not correspond to observed data (X^u, Z^u). We therefore introduce the following expanded notation:

$$X = \begin{bmatrix} X^o & X^u \end{bmatrix} \tag{43}$$

$$Z = \begin{bmatrix} Z^o & Z^u \end{bmatrix} \tag{44}$$

$$\Sigma = \begin{bmatrix} \Sigma^{oo} & (\Sigma^{uo})^T \\ \Sigma^{uo} & \Sigma^{uu} \end{bmatrix} \tag{45}$$

$$\Lambda = \begin{bmatrix} \Lambda^o & \Lambda^u \end{bmatrix} \tag{46}$$

$$\Gamma = \begin{bmatrix} \Gamma^{oo} & (\Gamma^{uo})^T \\ \Gamma^{uo} & \Gamma^{uu} \end{bmatrix}. \tag{47}$$

Additionally, let $P = P_o + P_u$ and $N = N_o + N_u$ denote the number of dimensions of the observed and unobserved sets, *i.e.*, Σ^{uo} is a $P_u \times P_o$ matrix and Γ^{ou} is an $N_u \times N_o$ matrix.

Proposition 5 *The GMGP models defined by Equations* (17)-(21) *are Marginally LTP models.*

Proof The proof of this proposition follows directly from Proposition 3 noting that the finite evaluation of a GMGP model on any finite sets $X^o = (X_{\cdot 1}, \dots, X_{\cdot N^o})$ and $Z^o = (Z_{\cdot 1}, \dots, Z_{\cdot P_o})$ can be written as a GMCL model given the following identifications: $\upsilon^o = \nu + P_o$, $\Xi^{oo} = \Xi(Z^o)$, $\Sigma^{oo} = \mathsf{\Sigma}(Z^o)$ $\Gamma^{oo} = \mathsf{\Gamma}(X^o)$, $\Theta^o = \mathsf{\Theta}(X^o)$, and $\Lambda^o = \mathsf{\Lambda}(X^o)$. With these identifications the GMGP model reduces to the following GMCL model

$$\begin{aligned} Y_{\cdot j} &\sim f(\pi_{\cdot j}) \\ \pi_{\cdot j} &= \phi^{-1}(\eta_{\cdot j}) \\ \eta_{\cdot j} &\sim N(\Lambda^o I_N, \Sigma^{oo}) \\ \Lambda^o &\sim N(\Theta^o, \Sigma^{oo}, \Gamma^{oo}) \\ \Sigma^{oo} &\sim IW(\Xi^{oo}, \upsilon^o) \end{aligned}$$

which – by Proposition 3 – is a Marginally LTP model. ■

Based on the identifications provided in the above proposition, it is straightforward to develop an efficient means of sampling $p(\Lambda^o, \Sigma^{oo} | \eta, Y, X^o, Z^o)$ (uncollapsing the GMGP model):

$$\begin{aligned} \upsilon^o_{N_o} &= \nu + P^o + N_o \\ \Gamma^{oo}_{N_o} &= (I + (\Gamma^{oo})^{-1})^{-1} \\ \Lambda^o_{N_o} &= (\eta + \Theta^o\, (\Gamma^{oo})^{-1}) \Gamma^{oo}_{N_o} \\ \Xi^{oo}_{N_o} &= \Xi^{oo} + (\eta - \Lambda^o_{N_o})(\eta - \Lambda^o_{N_o})^T + (\Lambda^o_{N_o} - \Theta^o)\, (\Gamma^{oo})^{-1}\, (\Lambda^o_{N_o} - \Theta^o)^T \\ p(\Sigma^{oo} | \eta, X) &= IW(\Xi^{oo}_{N_o}, \upsilon^o_{N_o}) \\ p(\Lambda^o | \Sigma^{oo}, \eta, X) &= N(\Lambda^o_{N_o}, \Sigma^{oo}, \Gamma^{oo}_{N_o}). \end{aligned}$$

So far we have described GMGP models for inferring the value of the latent stochastic processes $\mathsf{\Lambda}$ and $\mathsf{\Sigma}$ on the finite set corresponding to observed data (smoothing). Next we

address the challenge of sampling from the posterior distribution of the latent stochastic process over a finite set corresponding to both observed and unobserved points (simultaneously smoothing and predicting).

As described above, the CU sampler can be used to produce samples of $p(\Lambda^o, \Sigma^{oo}|\eta, Y, X^o, Z^o)$. Conditioned on those samples we now describe a method of sampling $p(\Lambda, \Sigma|X^o, Z^o, Y, X^u, Z^u)$. Letting $\Sigma^{ou/oo} = (\Sigma^{oo})^{-1}(\Sigma^{uo})^T$ and $\Sigma^{uu\cdot oo} = \Sigma^{uu} - \Sigma^{uo}\Sigma^{ou/oo}$, and using the conditional properties of the inverse Wishart distribution (Gupta and Nagar, 2018, pg. 112), we can sample Σ conditioned on Σ^{oo}:

$$\begin{aligned}
\Sigma^{uu\cdot oo} &\sim IW(\Xi^{uu\cdot oo}, \nu + p_o + p_u) \\
\Sigma^{ou/oo}|\Sigma^{uu\cdot oo} &\sim N(\Xi^{ou/oo}, (\Xi^{oo})^{-1}, \Sigma^{uu\cdot oo}) \\
\Sigma &= \begin{bmatrix} (\Sigma^{oo})^{-1} + \Sigma^{ou/oo}(\Sigma^{uu\cdot oo})^{-1}\left(\Sigma^{ou/oo}\right)^T & -\Sigma^{ou/oo}(\Sigma^{uu\cdot oo})^{-1} \\ -(\Sigma^{uu\cdot oo})^{-1}\left(\Sigma^{ou/oo}\right)^T & (\Sigma^{uu\cdot oo})^{-1} \end{bmatrix}.
\end{aligned}$$

Finally, we can sample Λ^u conditioned on Λ^o and Σ:

$$\begin{aligned}
\Gamma^{ou/oo} &= (\Gamma^{oo})^{-1}\Gamma^{ou} \\
\Gamma^{uu\cdot oo} &= \Gamma^{uu} - \Gamma^{uo}\Gamma^{ou/oo} \\
M &= \Theta^u + (\Lambda^o - \Theta^o)\Gamma^{ou/oo} \\
\Lambda^u|\Lambda^o, \Sigma &\sim N(M, \Sigma, \Gamma^{uu\cdot oo}).
\end{aligned}$$

Appendix D. Gradient and Hessian Calculations for the Matrix-T Distribution

Here we are concerned with calculating the gradient and Hessian of

$$\log p(\eta) \propto -\frac{\upsilon + N + P - 1}{2}\log\left|I_P + K^{-1}(\eta - B)A^{-1}(\eta - B)^T\right|.$$

Letting $S = I_P + K^{-1}(\eta - B)A^{-1}(\eta - B)^T$ we concern ourselves with calculating the quantities $\frac{d\log|S|}{d\mathrm{vec}(\eta^T)}$ and $\frac{d\log|S|}{\mathrm{vec}(d\eta)\mathrm{vec}(d\eta)^T}$. We will use the identity $d\log|S| = Tr(S^{-1}dS)$ from matrix calculus (Minka, 2000, pg. 1):

$$\begin{aligned}
d\log|S| &= Tr(S^{-1}dS) \\
dS &= d(I_P + K^{-1}(\eta - B)A^{-1}(\eta - B)^T) \\
&= d(K^{-1}(\eta A^{-1}\eta^T - \eta A^{-1}B^T - BA^{-1}\eta^T)) \\
&= K^{-1}(d\eta A^{-1}\eta^T + \eta A^{-1}d\eta^T - d\eta A^{-1}B^T - BA^{-1}d\eta^T) \\
&= K^{-1}(d\eta(A^{-1}\eta^T - A^{-1}B^T) + (\eta A^{-1} - BA^{-1})d\eta^T) \\
&= K^{-1}(d\eta C + C^T d\eta^T)
\end{aligned}$$

where in the last line we have defined the $N \times P$ matrix $C = A^{-1}(\eta^T - B^T)$. Further simplifying and using the identities $Tr(A) = Tr(A^T)$ and $Tr(AB) = Tr(BA)$ for matrices A and B we get

$$d\log|S| = Tr(S^{-1}K^{-1}(d\eta C + C^T d\eta^T))$$

$$
\begin{aligned}
&= Tr(S^{-1}K^{-1}d\eta C) + Tr(S^{-1}K^{-1}C^T d\eta^T) \\
&= Tr(CS^{-1}K^{-1}d\eta) + Tr(CK^{-1}S^{-T}d\eta) \\
&= Tr(C(S^{-1}K^{-1} + K^{-1}S^{-T})d\eta) \\
&= \text{vec}([C(S^{-1}K^{-1} + K^{-1}S^{-T})]^T)^T \text{vec}(d\eta) \\
d\log|S| &= \text{vec}((S^{-1}K^{-1} + K^{-1}S^{-T})C^T)^T \text{vec}(d\eta) \qquad (48) \\
\frac{d\log|S|}{\text{vec}(d\eta)} &= \text{vec}((S^{-1}K^{-1} + K^{-1}S^{-T})C^T)^T
\end{aligned}
$$

The Hessian $H = \frac{d^2\log|S|}{\text{vec}(d\eta)\text{vec}(d\eta)^T}$ can then be calculated from equation (48) by taking the differential again and manipulating the result into the following canonical form $d^2\log|S| = \text{vec}(d\eta)^T H \text{vec}(d\eta)$. In particular we make use of the following identities $\text{vec}(ABC) = (C^T \otimes A)\text{vec}(B)$ and $d(S^{-1}) = -S^{-1}dSS^{-1}$. We also make use of the vec-transposition matrix defined by $T_{m,n}\text{vec}(A) = \text{vec}(A^T)$ where A is an $m \times n$ matrix and $T_{m,n}$ is an $mn \times mn$ permutation matrix. The vec-transposition matrix also satisfies the following properties $T_{m,n} = T_{n,m}^T = T_{n,m}^{-1}$. Therefore we can write:

$$
\begin{aligned}
d^2\log|S| &= \text{vec}((S^{-1}K^{-1} + K^{-1}S^{-T})dC^T + d(S^{-1})K^{-1}C^T + K^{-1}d(S^{-T})C^T)^T \text{vec}(d\eta) \\
&= \left[\text{vec}((S^{-1}K^{-1} + K^{-1}S^{-T})dC^T)^T + \text{vec}(d(S^{-1})K^{-1}C^T)^T + \text{vec}(K^{-1}d(S^{-T})C^T)^T\right] \text{vec}(d\eta) \\
&= [\#1 + \#2 + \#3]\,\text{vec}(d\eta) \\
\#1 &= \text{vec}((S^{-1}K^{-1} + K^{-1}S^{-T})d\eta A^{-1})^T \\
&= ((A^{-1} \otimes (S^{-1}K^{-1} + K^{-1}S^{-T}))\text{vec}(d\eta))^T \\
&= \text{vec}(d\eta)^T (A^{-1} \otimes (S^{-1}K^{-1} + K^{-1}S^{-T}))^T \\
\#2 &= -\text{vec}(S^{-1}dSS^{-1}K^{-1}C^T)^T \\
&= -((CK^{-1}S^{-T} \otimes S^{-1})\text{vec}(dS))^T \\
&= -\text{vec}(dS)^T (S^{-1}K^{-1}C^T \otimes S^{-T}) \\
\text{vec}(dS)^T &= \text{vec}(K^{-1}(d\eta C + C^T d\eta^T))^T \\
&= \text{vec}(K^{-1}d\eta C)^T + \text{vec}(K^{-1}C^T d\eta^T)^T \\
&= ((C^T \otimes K^{-1})\text{vec}(d\eta))^T + ((I_{D-1} \otimes K^{-1}C^T)\text{vec}(d\eta^T))^T \\
&= \text{vec}(d\eta)^T (C \otimes K^{-1}) + \text{vec}(d\eta^T)^T (I_P \otimes CK^{-1}) \\
\#2 &= [-\text{vec}(d\eta)^T (C \otimes K^{-1}) - \text{vec}(d\eta^T)^T (I_P \otimes CK^{-1})](S^{-1}K^{-1}C^T \otimes S^{-T}) \\
&= -\text{vec}(d\eta)^T (CS^{-1}K^{-1}C^T \otimes K^{-1}S^{-T}) - \text{vec}(d\eta^T)^T (S^{-1}K^{-1}C^T \otimes CK^{-1}S^{-T}) \\
&= -\text{vec}(d\eta)^T (CS^{-1}K^{-1}C^T \otimes K^{-1}S^{-T}) - \text{vec}(d\eta)^T T_{N,P} (S^{-1}K^{-1}C^T \otimes CK^{-1}S^{-T}) \\
\#3 &= \text{vec}(K^{-1}d(S^{-T})C^T)^T \\
&= -\text{vec}(K^{-1}S^{-T}dS^T S^{-T}C^T) \\
&= -((CS^{-1} \otimes K^{-1}S^{-T})\text{vec}(dS^T))^T \\
&= -\text{vec}(dS^T)^T (S^{-T}C^T \otimes S^{-1}K^{-1}) \\
\text{vec}(dS^T)^T &= \text{vec}((d\eta C + C^T d\eta^T)^T K^{-1})^T
\end{aligned}
$$

$$
\begin{aligned}
&= ((K^{-1}C^T \otimes I_P)\text{vec}(d\eta))^T + ((K^{-1} \otimes C^T)\text{vec}(d\eta^T))^T \\
\#3 &= [-\text{vec}(d\eta)^T(CK^{-1} \otimes I_P) - \text{vec}(d\eta^T)^T(K^{-1} \otimes C)](S^{-T}C^T \otimes S^{-1}K^{-1}) \\
&= -\text{vec}(d\eta)^T(CK^{-1}S^{-T}C^T \otimes S^{-1}K^{-1}) - \text{vec}(d\eta)^T T_{N,D-1}(K^{-1}S^{-T}C^T \otimes CS^{-1}K^{-1}) \\
d^2 \log|S| &= \text{vec}(d\eta)^T[(A^{-1} \otimes (S^{-1}K^{-1} + K^{-1}S^{-T}))^T - (CS^{-1}K^{-1}C^T \otimes K^{-1}S^{-T}) \\
&\quad - (CK^{-1}S^{-T}C^T) \otimes S^{-1}K^{-1}) \\
&\quad - T_{N,D-1}((S^{-1}K^{-1}C^T \otimes CK^{-1}S^{-T}) + (K^{-1}S^{-T}C^T \otimes CS^{-1}K^{-1}))]\text{vec}(d\eta)
\end{aligned}
$$

Summarizing the above results we obtain

$$
\begin{aligned}
S &= I_P + K^{-1}(\eta - B)A^{-1}(\eta - B)^T \\
C &= A^{-1}(\eta - B)^T \\
R &= S^{-1}K^{-1} \\
\frac{d\log|S|}{\text{vec}(d\eta)} &= \text{vec}((R + R^T)C^T)^T \\
L &= (CRC^T \otimes R^T) \\
\frac{d^2\log|S|}{\text{vec}(d\eta)\text{vec}(d\eta)^T} &= (A^{-1} \otimes (R + R^T)) - (L + L^T) - T_{N,D-1}[(RC^T \otimes CR^T) + (R^TC^T \otimes CR)].
\end{aligned}
$$

Finally, we note a computational trick which makes evaluation of this Hessian far more computationally efficient. We may quickly calculate $T_{m,n}X = X^*$ for an $m \times m$ matrix X having already computed X by noting that for $i \in 1 \dots m$ and $j \in 1 \dots n$ we can write $X^*_{(i-1)n+j,\cdot} = X_{(j-1)m+i,\cdot}$ where $X_{l,\cdot}$ denotes the l-th row of the matrix X.

Appendix E. Gradients and Hessians for the Log-Ratio Parameterized Multinomial

Unfortunately we cannot provide a general form for the gradient and Hessian of all possible likelihoods $f(Y \mid \phi^{-1}(\eta))$. For the purposes of this article, here we derive the gradient and Hessian for the case where f is multinomial and ϕ^{-1} is the inverse ALR transform:

$$
\sum_j \log \text{Multinomial}(Y_{\cdot j} \mid n_j, \text{ALR}_D^{-1}(\eta_{\cdot j}))
$$

which for notational simplicity we will refer to as g. Thus our goal is to find efficient forms for calculating g, $\frac{dg}{d\text{vec}(\eta)}$ and $\frac{d^2 g}{d\text{vec}(\eta)d\text{vec}(\eta)^T}$. Using the fact that $\log \text{Multinomial}(Y_{\cdot j} \mid n_j, \pi_{\cdot j}) \propto Y_{1j}\log\pi_{1j} + \cdots + Y_{Dj}\log\pi_{Dj}$ and Equation (1) we can write

$$
g = \sum_{j=1}^{N}\left(\sum_{i=1}^{D-1}\eta_{ij}Y_{ij} - n_j\log\left(1 + \sum_{i=1}^{D-1}e^{\eta_{ij}}\right)\right).
$$

Differentiating with respect to η_{ij} gives

$$
\frac{dg}{d\eta_{ij}} = Y_{ij} - n_j\frac{e^{\eta_{ij}}}{1 + \sum_i e^{\eta_{ij}}}.
$$

Differentiating again with respect to $\eta_{k\ell}$ gives

$$\frac{d^2 g}{d\eta_{ij} d\eta_{k\ell}} = \begin{cases} -n_j \left(\frac{e^{\eta_{ij}}}{1+\sum_i e^{\eta_{ij}}} - \frac{e^{2\eta_{ij}}}{(1+\sum_i e^{\eta_{ij}})^2}\right) & \text{if } \ell = j, i = k \\ n_j \left(\frac{e^{\eta_{ij}} e^{\eta_{kj}}}{(1+\sum_i e^{\eta_{ij}})^2}\right) & \text{if } \ell = j, i \neq k \\ 0 & \text{if } \ell \neq j. \end{cases}$$

These results directly imply the following matrix forms.

$$\begin{aligned} O &= \exp \eta \\ m &= 1_N + O^T 1_{D-1} \\ \rho &= \text{vec}(O) \oslash \text{vec}(1_{D-1} m^T) \\ n &= 1_D^T Y \\ g &= -\text{vec}(\eta)^T \text{vec}(Y_{/D\cdot}) - n \odot \log(m) \\ \frac{dg}{d\text{vec}(\eta)} &= (\text{vec}(Y_{/D\cdot}) - \text{vec}(1_D n) \odot \rho)^T \\ W^{(j)} &= n_j (\rho_{(j)} \rho_{(j)}^T - \text{diag}(\rho_{(j)})) \\ \frac{d^2 g}{d\text{vec}(\eta) d\text{vec}(\eta)^T} &= \text{diag}\left(W^{(1)}, \ldots, W^{(N)}\right) \end{aligned}$$

where $\exp X$ and $\log X$ refers to the element-wise exponentiation and logarithm of a matrix X, $\odot$ and $\oslash$ refer to element-wise product and division respectively, $Y_{/D\cdot}$ refers to the first $D-1$ rows of the matrix Y, $\rho_{(j)}$ denotes elements $(j-1)(D-1)+1$ to $j(D-1)$ in the vector ρ, and $\text{diag}(X_1, \ldots, X_D)$ refers to a block diagonal matrix where the i-th block is X_i.

Appendix F. Implementing the Laplace Approximation to an LTP

Implementing this Laplace approximation for an LTP requires three steps: finding the MAP estimates for η using optimization; calculating the hessian at the MAP estimate, and then sampling from the approximating normal distribution.

F.1 Finding the MAP estimate

The MAP estimate for η (denoted $\hat{\eta}$) is defined as the solution to the following optimization problem:

$$\hat{\eta} = \underset{\eta \in \mathcal{R}^{P \times N}}{\text{argmin}} \left[-\log p(\eta \mid Y)\right] \tag{49}$$

where $\log p(\eta|Y)$ is the sum of the log-matrix-t prior and log-likelihood densities as shown in Equation (23):

$$-\log p(\eta \mid Y) \propto -\log f(Y \mid \phi^{-1}(\eta)) - p(\eta).$$

The form of $p(\eta)$ is given in Appendix D. In contrast, the form of $\log f(Y \mid \phi^{-1}(\eta))$ depends on the choice of likelihood (f) and link function (ϕ). When f and ϕ are given respectively by the multinomial and ALR transformation, the resulting form of $\log f(Y \mid \phi^{-1}(\eta))$ can be found in Appendix E.

As we expect the dimension of η to be large in most applications, we recommend using gradient based optimization methods such as L-BFGS over methods that require repeated calculation or inversion of a hessian matrix such as Newton-Raphson (Sun et al., 2019). Beyond calculating $\log p(\eta \mid Y)$, gradient based optimization additionally requires calculating the gradient $-\frac{d \log p(\eta|Y)}{d\text{vec}(\eta)}$, which is given by Equation (24):

$$-\frac{d \log p(\eta \mid Y)}{d\text{vec}(\eta)} = -\frac{d \log f(Y \mid \phi^{-1}(\eta))}{d\text{vec}(\eta)} - \frac{d \log p(\eta)}{d\text{vec}(\eta)}.$$

The form of $\frac{d \log p(\eta)}{d\text{vec}(\eta)}$ is given in Appendix D. For added computational efficiency when $N < P$, we provide an alternative method of calculating the gradient $\frac{d \log p(\eta)}{d\text{vec}(\eta)}$ using Sylvester's determinant identity in Appendix G. In contrast, the form of $\frac{d \log f(Y|\phi^{-1}(\eta))}{d\text{vec}(\eta)}$ depends on the choice of likelihood (f) and link function (ϕ). When f and ϕ are given respectively by the multinomial and ALR transformation, the resulting form of $\frac{d \log f(Y|\phi^{-1}(\eta))}{d\text{vec}(\eta)}$ can be found in Appendix E.

F.2 Calculating the hessian at the MAP estimate

Once the MAP estimate $\hat{\eta}$ has been found, the hessian H at the MAP estimate (denoted $H(\text{vec}\,\hat{\eta})$ can be calculated using Equation (25):

$$H = \frac{d^2 \log f(Y \mid \phi^{-1}(\eta))}{d\text{vec}(\eta) d\text{vec}(\eta)^T} + \frac{d^2 \log p(\eta)}{d\text{vec}(\eta) d\text{vec}(\eta)^T}.$$

The form of $\frac{d^2 \log f(Y|\phi^{-1}(\eta))}{d\text{vec}(\eta) d\text{vec}(\eta)^T}$ is given in Appendix D. In contrast, the form of $\frac{d^2 \log f(Y|\phi^{-1}(\eta))}{d\text{vec}(\eta) d\text{vec}(\eta)^T}$ depends on the choice of likelihood (f) and link function (ϕ). When f and ϕ are given respectively by the multinomial and ALR transformation, the resulting form of $\frac{d^2 \log f(Y|\phi^{-1}(\eta))}{d\text{vec}(\eta) d\text{vec}(\eta)^T}$ can be found in Appendix E.

F.3 Sampling from the approximating normal distribution

The Laplace approximation to the density $p(\eta|Y)$ is defined as $q(\eta|Y) = N(\text{vec}\,\hat{\eta}, H^{-1}(\text{vec}\,\hat{\eta}))$. While there are numerous ways of sampling from $q(\eta|Y)$ explicit inversion of H^{-1} can be avoided using a Cholesky decomposition. Letting U denote the upper Cholesky factor of the matrix $H^{-1}(\text{vec}\,\hat{\eta})$ such that $H^{-1}(\text{vec}\,\hat{\eta}) = U^T U$ we may sample a random variable $\text{vec}\,\eta^{(s)} \sim q(\eta|Y)$ by first sampling a vector of standard normal variables z and then transforming that sample as:

$$\text{vec}\,\eta^{(s)} = \text{vec}\,\hat{\eta} + U^{-1} z.$$

In practice, it is often more computationally efficient to directly calculate $U^{-1}z$ by backsolving rather than directly computing the term U^{-1}.

Appendix G. Accelerated Matrix-T Gradients via Sylvester's Determinant Identity

Sylvester's determinant identity states that for matrices A and B of size $m \times n$ and $n \times m$ respectively, $|I_m + AB| = |I_n + BA|$. This relationship can be used to speed up calculation

of the log-likelihood and gradient of the matrix-t distribution when $N < P$ as the the log determinant or inverse of the matrix S can dominate computational time. To take advantage of this speed up we note that we can replace the relations given in Appendix D with

$$\begin{aligned} S &= I_N + A^{-1}(\eta - B)^T K^{-1}(\eta - B) \\ C &= K^{-1}(\eta - B) \\ R &= S^{-1}A^{-1} \\ \frac{d \log |S|}{d\text{vec}(\eta)} &= \text{vec}(C(R + R^T))^T. \end{aligned}$$

While this result can greatly accelerate inference for matrix-t gradients when $P \gg N$, this result provides only minimal improvement for calculating the corresponding Hessian terms. Therefore we suggest that, for simplicity, the Hessian form provided in Appendix D be used even if $P \gg N$.

Appendix H. Simulations and Model Fitting

To compare the performance of the multiple multinomial logistic-normal linear model implementations described in Section 5 over a range of sample sizes (N), observation dimensions (D), and covariate dimensions (Q), we created the following simulation scheme. For each evaluated triple (N, D, Q), three simulated data-sets were created based on the multinomial logistic-normal linear model with the following specified likelihood:

$$\begin{aligned} Y_{\cdot j} &= \text{Multinomial}(n_j, \pi_{\cdot j}) \\ \pi_{\cdot j} &= \text{ALR}_D^{-1}(\eta_{\cdot j}) \\ \eta_{\cdot j} &= N(\Lambda X_{\cdot j}, \Sigma). \end{aligned}$$

Additionally X, Λ, and Σ were simulated as

$$\begin{aligned} \Lambda &\sim N(0, I, I) \\ \Sigma &\sim IW(I, D + 10) \\ X &\sim N(0, I, I). \end{aligned}$$

The percent of zero counts naturally increased with large D or large Q relative to other parameters. We took advantage of this behavior to study the performance of all implementations in sparse data regimes.

For all model fits, priors parameter values for υ, Ξ, and Θ and values for hyperparameter Γ were chosen as their simulated values. All implementations were compiled and run using gcc version 6.2.0, R version 3.4.2, and Intel(R) Math Kernel Library version 2019 where possible. All replicates of the simulated count data were supplied to the various implementations independently and the models were fit on identical hardware, allotted 64GB RAM, 4 cores, and restricted to a 48-hour upper limit on run-time.

Appendix I. Priors for Crohn's Disease Data

Sequence count data was obtained from the R package MicrobeDS (github.com/twbattaglia/MicrobeDS). Only samples from the terminal ileum from healthy donors and patients with Crohn's Disease, who had no recent history of steroids, antibiotics, or biologics were included in the analysis. Samples with a sequencing depth below 5000 counts were excluded from analyses. Only families seen with at least 3 counts in at least 10% of samples were retained for subsequent analyses.

The regression model required that 4 hyper-parameters Γ, Θ, Ξ, and υ be specified. We set Θ to a $D \times Q$ matrix of zeros representing our prior assumption that, on average, there was no association between each covarariate and microbial composition. We specified $\Gamma = I_Q$ to constrain associations between microbial composition and covariates to remain small. We specified $\upsilon = D + 3$ and $\Xi_{ij} = (\upsilon - D)$ if $i = j$ and $\Xi_{ij} = (\upsilon - D)/2$ if $i \neq j$ to reflect our weak prior assumption that the log absolute abundance of each taxa is uncorrelated (Aitchison, 1986, p. 208-214).

Appendix J. Priors for Artificial Gut Data

Sequence count data was obtained from the R package Fido (github.com/jsilve24/fido). Only samples from the high-resolution hourly sampling period were included in the analysis.

The the developed GMGP model required that 4 hyper-parameters be specified: Θ, $\mathsf{\Gamma}_{\text{time}}$,$\mathsf{\Gamma}_{\text{vessel}}$, Ξ, and ν. Per the default in *fido*, these hyper-parameters were specified with respect to ALR_D coordinates. We specified $\Theta = \mathbf{0}$ which centered our prior about the neutral element of the simplex. We specified $\mathsf{\Gamma}_{\text{time}}$ as a squared exponential kernel

$$\mathsf{\Gamma}_{\text{time}}(t_i, t_j) = \exp \frac{-|t_j - t_i|^2}{2\rho_t^2}$$

where ρ_t was set to the median temporal distance between samples. To induce independence between vessels, letting r_i denote the vessel sample i was taken from, we specified $\mathsf{\Gamma}_{\text{vessel}}$ as

$$\mathsf{\Gamma}_{\text{vessel}}(r_i, r_j) = \begin{cases} 1 \text{ if } r_i = r_j \\ 0 \text{ otherwise} \end{cases} .$$

Next, following prior reports, we assumed that more evolutionary similar bacterial taxa would behave more similarly (Silverman et al., 2017). We encoded this prior information in a phylogenetic kernel for Ξ as follows. Let h_{ij} denote the Hamming distance between the 16S sequence of taxa i (s_i) and j (s_j). We created a squared exponential kernel based on these distances:

$$\Xi^*(s_i, s_j) = \exp \frac{-h_{ij}^2}{2\rho_s^2}$$

where ρ_s was set as the median hamming distance between sequences. To project the kernel $\Xi^*(s_i, s_j)$ into ALR_D coordinates, we created a kernel Ξ which was specified as the projection of the Gram matrix of Ξ^*. Letting G denote the contrast matrix for ALR_D ($G = [I_{D-1}, -1]$) and letting Ξ^* represent the Gram matrix of the kernel Ξ^* and Ξ represent the Gram matrix of the kernel Ξ, we specified the kernel Ξ implicitly as

$$\Xi = \text{corr}(G\Xi^*G^T)$$

where corr represents the normalized correlation matrix corresponding to a symmetric positive definite matrix. Finally, we specified $\nu = D + 2$ to reflect the fact that our prior knowledge regarding Ξ was weak.

Appendix K. Laplace Approximation Error

In this section we provide an analysis of the error rate of Laplace approximation used in Section 4.2.

Given an integral of the form

$$L = \int_{R^d} e^{-g(u)} du \tag{50}$$

Ogden (2018) examined conditions and rates for order-k Laplace approximations of the above integral. There were two regularity and convexity conditions required in the analysis.

Condition 1 *$g(\cdot)$ is a smooth function with a unique minimum.*

Condition 2 *Denote the unique minimum of $g(\cdot)$ as $\hat{u}$ and H_{ij} as the ij-th element of the Hessian of g evaluated at $\hat{u}$. For a collection of normalizing terms $\alpha_1, ..., \alpha_d > 0$ the normalized derivatives are*

$$k_{ij} = \frac{h_{ij}}{\alpha_i^{1/2} \alpha_j^{1/2}}.$$

For a $d \times d$ matrix A we denote $A = O_p^*(1)$ if for each $i, j \in 1, ..., d$ $\sum_j |A_{ij}| = O_p(1)$ and $\sum_i |A_{ij}| = O_p(1)$.

The second condition is there exist normalizing terms $\alpha_1, ..., \alpha_d$ such that $k^{-1} = O_p^*(1)$.

The main result we will use is (Ogden, 2018, Theorem 1) which states if Condition 1 and 2 are met then the error rate of the order-1 Laplace approximation to L is given by $\epsilon = O_p(\sum_{j=1}^d \alpha_j^{-1})$ where α_j are determined by Condition 2.

To prove the error rate of our Laplace approximation we will need the following Lemmas.

Lemma 6 *Let $\lambda_{\min}(X)$ denote the minimum eigenvalue of a matrix X. Assuming A and B denote Hermitian matrices. Then $\lambda_{\min}(A + B) \geq \lambda_{\min}(A) + \lambda_{\min}(B)$.*

Proof H is Hermitian so all eigenvalues must be real. For a Hermitian matrix A and non-zero vector x we have $x^T Ax \geq \lambda_{\min}(A)x^T x$ such that $x^T Ax = \lambda_{\min}(A)x^T x$ if and only if $x \in \text{Span}(x_{\min}(A))$ where $x_{\min}(A)$ denotes the set of eigenvectors corresponding to the minimum eigenvalue of A. It therefore follows that

$$\begin{aligned} x^T(A + B)x &= x^T Ax + x^T Bx \\ &\geq (\lambda_{\min}(A) + \lambda_{\min}(B))x^T x \end{aligned}$$

and this minimum bound is achieved if and only if $x \in \text{Span}(x_{\min}(A)) \cap \text{Span}(x_{\min}(B))$. It follows that $\lambda_{\min}(A + B) \geq \lambda_{\min}(A) + \lambda_{\min}(B)$ and equality is achieved only if there exists an x such that $x \in \text{Span}(x_{\min}(A)) \cap \text{Span}(x_{\min}(B))$. ■

Lemma 7 *For Hermitian matrices A and B, $\lambda_{\min}(A \circ B) \geq \lambda_{min}(A)\lambda_{\min}(B)$ where $\circ$ denotes the Hadamard (element-wise) product.*

Proof The Hadamard product $A \circ B$ is a principle sub-matrix of the Kroneker product $A \otimes B$. We may define the principle sub-matrix using a selection matrix E such that we may write $A \circ B = E^T(A \otimes B)E$. Letting $A = UD_AU^T$ and $B = VD_BV^T$ denote the eigen-decompositions of A and B respectively, we can then write

$$\begin{aligned} A \circ B &= E^T(UD_AU^T \otimes VD_BV^T)E \\ &= E^T[(U \otimes V)(D_A \otimes D_B)(U \otimes V)^T]E \end{aligned}$$

Therefore the eigenvalues of $A \circ B$ represent of subset of the eigenvalues of $A \otimes B$. As the eigenvalues of $A \otimes B$ are given by every pairwise product between one eigenvalue of A and one eigenvalue of B it is therefore clear that the minimum eigenvalue of $A \otimes B$ is $\lambda_{\min}(A)\lambda_{\min}(B)$. Therefore $\lambda_{\min}(A \circ B) \geq \lambda_{\min}(A)\lambda_{\min}(B)$. ∎

Lemma 8 *For a $d \times d$ symmetric positive definite matrix H, $H = O_p^*(1)$ if $\lambda_{max}(H) = O_p(1)$ where $\lambda_{max}(.)$ denotes the maximum eigenvalue operator.*

Proof If H is symmetric then $H = H^T$ and therefore $\sum_j |H_{ij}| = O_p(1)$ for all $j \in \{1, \ldots, d\}$ if and only if $\sum_i |H_{ij}| = O_p(1)$ for all $i \in \{1, \ldots, d\}$. For a vector x we have $x^THx \leq \lambda_{\max}(H)x^Tx$ with equality only if $x \in \text{Span}(x_{\max}(H))$ where $x_{\max}(H)$ denotes the set of eigenvectors corresponding $\lambda_{\max}(H)$. Letting r denote a D-vector with elements defined by $r_j = \sum_j H_{ij}$ it is clear that $r = H1_D$. Therefore, $r^Tr = 1_d^THH1_d \leq d\lambda_{\max}^2(H)$. Thus $r^Tr \leq d\lambda_{\max}^2(H)$. As d is a constant and given that $\lambda_{\max}(H) = O_p(1)$ it follows that $r^Tr = O_p(1)$. Noting that $r^Tr = \sum_i \sum_j |H_{ij}|^2$ we can conclude that $\sum_j |H_{ij}| < r^Tr$ for all $j \in \{1, \ldots, D\}$. Therefore since $r^Tr = O_p(1)$ we must have that $\sum_j |H_{ij}| = O_p(1)$ for all j and therefore, by definition, that $H = O_p^*(1)$. ∎

Lemma 9 *For a $d \times d$ symmetric positive definite matrix H, if $\lambda_{\min}(H) = \Omega_p(1)$ then $H^{-1} = O_p^*(1)$, where $\Omega_p(1)$ a stochastic lower-bound of order at least 1.*

Proof Denoting the eigen-decomposition of H as $H = VDV^T$ we can write $H^{-1} = VD^{-1}V^T$ where D^{-1} is a diagonal matrix with elements $D_{ii}^{-1} = 1/D_{ii}$. It follows then $\lambda_{\max}(H^{-1}) = \lambda_{\min}(H)^{-1}$. If H is symmetric positive definite then all eigenvalues of H are positive. Therefore if $\lambda_{\min}(H)$ is lower bounded such that $\lambda_{\min}(H) = \Omega_p(1)$ then we can conclude that $\lambda_{\max}(H^{-1})$ is upper bounded by $\lambda_{\max}(H^{-1}) = O_p(1)$. Using Lemma 8 it follows that $H^{-1} = O_p^*(1)$. ∎

Lemma 10 *The function $g(\eta) = \sum_j \log Multinomial(Y_{\cdot j}|n_j, ALR_D^{-1}(\eta_{\cdot j}))$ is strictly concave.*

Proof Let g_j denote the j-th element in the sum such that

$$g_j(\eta_{\cdot j}) = \log \text{Multinomial}(Y_{\cdot j} | n_j, \text{ALR}_D^{-1}(\eta_{\cdot j})).$$

g_j can then be equivalently written as

$$g_j(\eta_{\cdot j}) = \sum_{i=1}^{D-1} \eta_{ij} Y_{ij} - n_j \log \left(1 + \sum_{i=1}^{D-1} e^{\eta_{ij}} \right). \tag{51}$$

As the sum of concave functions is itself concave, our proof relies on showing that each g_j is concave.

Denoting the natural exponential family as $\log p(x|\gamma) \propto \gamma \cdot T(x) - A(\gamma)$ we can see that g_j corresponds to a natural exponential family density with natural parameters $\gamma = \eta$, sufficient statistic $T(x) = Y_{\cdot j}$ and log-partition function $A(\gamma) = n_j \log \left(1 + \sum_{i=1}^{D-1} e^{\eta_{ij}}\right)$. The hessian $\log p(x|\gamma)$ is

$$\frac{d^2 \log p(x|\gamma)}{d\gamma_i d\gamma_j} = -\frac{d^2 A(\gamma)}{d\gamma_i d\gamma_j}.$$

Furthermore, for all natural exponential family densities, the log-partition function $A(\eta)$ is strictly convex Jordan (2010). Therefore the hessian of g_j with elements $d^2 g_j(\eta_{\cdot j})/d\eta_{ij} d\eta_{kj}$ is positive definite for all values of $\eta_{\cdot j}$ and we can therefore conclude that g_j is strictly concave. Since g is the sum of strictly concave function we can conclude that g is strictly concave. ■

Proposition 11 *Let $\mathcal{Y}$ denote the finite realization of a D-dimensional LTP evaluated on an $N \times (D-1)$ finite set such that $\mathcal{Y}$ has the following form:*

$$\begin{aligned} Y_{\cdot j} &\sim \textit{Multinomial}(n_j, \pi_j) \\ \pi_{\cdot j} &= ALR_D^{-1}(\eta_{\cdot j}) \\ \eta &\sim T(\upsilon, B(\delta), K(\delta), A(\delta)). \end{aligned} \tag{52}$$

Assuming that $A(\delta)$ and $K(\delta)$ are symmetric positive definite and do not vary with any n_j. In the limit as $\upsilon \to \infty$ the error for the order-1 Laplace approximation to $\int p_{\mathcal{Y}}(Y, \eta) d\eta$ is $\epsilon = O_p((D-1) \sum_{j=1}^{N} n_j^{-1})$.

Proof Without loss of generality we may redefine $K(\delta) \to \upsilon K(\delta)$ such that (52) can be written as $\eta \sim T(\upsilon, B(\delta), \upsilon K(\delta), A(\delta))$. Given such a form, Theorem 4.3.4 of Gupta and Nagar (2018) proves that as $\upsilon \to \infty$, η converges in distribution to $\eta \sim N(B(\delta), K(\delta), A(\delta))$ such that in the limit we may write $\mathcal{Y}$ as

$$\begin{aligned} Y_i &\sim \text{Multinomial}(n_i, \pi_i) \\ \pi_i &= ALR_D^{-1}(\eta_i) \\ \eta &\sim N(B(\delta), K(\delta), A(\delta)). \end{aligned}$$

In this limit, we may write

$$\begin{aligned}\int p_{\mathcal{Y}}(Y,\eta)d\eta &= \int p(\eta)p(Y|\eta)d\eta \\ &= \int N(\eta|B(\delta),K(\delta),A(\delta))\prod_j \text{Multinomial}(Y_{\cdot j}|n_j,\text{ALR}_D^{-1}(\eta_{\cdot j}))d\eta \\ &= \int \exp\left\{-\log N(\eta|B(\delta),K(\delta),A(\delta)) - \sum_j \log\text{Multinomial}(Y_{\cdot j}|n_j,\text{ALR}_D^{-1}(\eta_{\cdot j}))\right\}d\eta \\ &= \int_{R^{P(D-1)}} \exp\{-g(\eta)\}d\eta\end{aligned}$$

The above integral therefore has the form studied by Ogden (2018) and our goal is to prove that Conditions 1 and 2 hold for some choice of normalizing constants $\alpha_1,\dots,\alpha_{P(D-1)}$.

To show that Condition 1 holds, we must show that $g(\eta)$ is smooth with unique optima. $g(\eta)$ can be represented as a sum $g(\eta) = -(g_N(\eta) + g_M(\eta))$ where

$$\begin{aligned}g_N(\eta) &= \log N(\eta|B(\delta),K(\delta),A(\delta)) \\ g_M(\eta) &= \sum_j \log\text{Multinomial}(Y_{\cdot j}|n_j,\text{ALR}_D^{-1}(\eta_{\cdot j})).\end{aligned}$$

It is clear that $g(\eta)$ is smooth for all $\epsilon \in R^{P(D-1)}$. We prove that $g(\eta)$ has a unique optima by showing that $g(\eta)$ is strictly convex. As $K(\delta)$ and $A(\delta)$ are positive definite it follows from the properties of the matrix-normal that $g_N(\eta)$ is strictly concave. Furthermore, in Lemma 10 we proved that $g_M(\eta)$ is strictly concave. Therefore $g(\eta)$ is the sum of two strictly convex functions and is therefore strictly convex. As $g(\eta)$ is strictly convex it therefore has a single unique optima.

To show that Condition 2 holds we chose normalizing constants $\alpha_{i\times j}$ for $i \in \{1,\dots,P\}$ and $j \in \{1,\dots,D-1\}$ such that $\alpha_{i\times j} = n_j$. We do this by bounding the minimum eigenvalue of the Hessian $h = g''(\hat{\eta})$ and using Lemma 9. Based on the linearity of the derivative operator we can write $h = -(h_N + h_M)$ where h_N and h_M are defined as the Hessian of g_N and g_M respectively evaluated at the optima $\hat{\eta}$. If $A(\delta)$ and $K(\delta)$ do not depend on $n_1,\dots,n_j$ then g_N and therefore h_N has no dependence on $n_1,\dots,n_j$. Noting that $h_N = A(\delta)\otimes K(\delta)$ we can then write

$$k_N = (A(\delta)\otimes K(\delta)) \oslash (\alpha\otimes\alpha^T)$$

where $\oslash$ denotes Hadamard (element-wise) division and α denotes the vector of normalizing constants. Note that $\alpha\otimes\alpha^T$ is strictly positive rank-1 matrix and therefore $\lambda_{\min}(\alpha\otimes\alpha^T) \geq 0$. Similarly, since $A(\delta)$ and $K(\delta)$ are both symmetric positive definite we have that $\lambda_{\min}(A(\delta)\otimes K(\delta)) \geq 0$. Noting that $A \oslash B = A \circ (1 \oslash B)$ we can use Lemma 7 to conclude that $\lambda_{min}(k_N) \geq 0$. Moving onto h_M, we use results in Appendix E to represent h_M as a block diagonal matrix

$$h_M = \text{diag}\left(n_1C^{(1)},\dots,n_NC^{(N)}\right).$$

It follows that

$$k_M = \text{diag}\left(C^{(1)},\dots,C^{(N)}\right)$$

where the blocks $C^{(j)}$ are $(D-1)\times(D-1)$ symmetric positive definite matrices of full rank. Therefore the minimum eigenvalue of k_M is greater than zero and does not vary with $n_1,\dots,n_N$. We now have shown that $\lambda_{\min}(k_N)\geq 0$ and $\lambda_{\min}(k_H)>0$, the latter we have also shown has no dependence on $n_1,\dots,n_N$. Combining these results with Lemma 6 we can conclude that $\lambda_{\min}(k)\geq c>0$ where c is a constant defined by $c=\lambda_{\min}(k_M)$. It follows that $\lambda_{\min}(k)=\Omega_p(c)=\Omega_p(1)$ and therefore from Lemma 9 that $k^{-1}=O_p^*(1)$.

We now have shown that $\int p_{\mathcal{Y}}(Y,\eta)d\eta$ is of the form (50), that Condition 1 and Condition 2 hold with normalizing constants $\alpha_{i\times j}$ for $i\in\{1,\dots,P\}$ and $j\in\{1,\dots,D-1\}$ such that $\alpha_{i\times j}=n_j$. Therefore we have $\epsilon=O_p((D-1)\sum_{j=1}^{N}n_j^{-1})$. ■
